# Comprehensive identification of glutathione peroxidase (GPX) gene family and effect of GhGPX4 on reactive oxygen species metabolism in cotton

**DOI:** 10.3389/fpls.2026.1846132

**Published:** 2026-06-26

**Authors:** Li Zhang, Jinlong Zhang, Yuekai Su, Jinjiang Shi, Yanqiong Guo, Jinling Huang, Yunfang Qu

**Affiliations:** 1 College of Agriculture, Shanxi Agricultural University, Jinzhong, Shanxi, China; 2 College of Plant Protection, Shanxi Agricultural University, Jinzhong, Shanxi, China; 3 College of Forestry, Shanxi Agricultural University, Jinzhong, Shanxi, China

**Keywords:** glutathione, glutathione peroxidase, glutathione S-transferase, interaction protein, reactive oxygen species, thioredoxin

## Abstract

Plant glutathione peroxidase (GPX) is an antioxidant enzyme that uses thioredoxin as a reductant. GPX plays key roles in modulating the metabolism of reactive oxygen species (ROS). In this study, expression pattern analysis of *GPX* in *Gossypium hirsutum* (cotton) revealed that *GhGPX4* and *GhGPX12* were responsive to low temperature, drought, and salt stress. ROS levels were significantly increased in *GhGPX4*-silenced cotton, whereas antioxidant enzyme activity decreased. *GhGPX4* overexpression in *Arabidopsis* flowers and leaves reduced ROS levels, thus indicating the ROS-scavenging function of chloroplast GhGPX4. Our findings indicate that GhGPX4 migrates to both the cytoplasm and nucleus, and executes its physiological functions through physical interactions with glutathione S-transferase. These results provide a theoretical foundation for further investigation into the involvement of GPX in redox reactions and the regulation of plant ROS metabolism.

## 1 Introduction

Reactive oxygen species (ROS), including hydrogen peroxide (H_2_O_2_), phospholipid hydroperoxides, superoxide radicals (O_2_^^-^•^), and singlet oxygen (^1^O_2_), are naturally generated byproducts of various cellular metabolic reactions. These molecules play essential roles in cell signal transduction and the regulation of redox homeostasis. Under certain conditions, excessive ROS accumulation in plants occurs due to an increase in ROS production or inhibition of the defence system. This imbalance in ROS production leads to the oxidative damage of macromolecules, such as DNA, proteins, and lipids, resulting in irreversible physiological damage. To cope with ROS instability in cells, plants have evolved complex antioxidant systems containing various antioxidant enzymes and metabolites in different tissues, cells, and organelles. In plants, fluctuations in environmental conditions, including light intensity and temperature, modify electron flow within chloroplasts and mitochondria, whilst the ROS scavenging is subject to stringent regulation.

The generation and scavenging of chloroplasts ROS depend on a sustained light-driven electron flux and NADPH and accompany redox cascade reactions by thioredoxin (TRX) and glutathione (GSH) (Meyer et al., 2012; Yoshida and Hisabori, 2016). Under oxidative stress conditions, the increasing activities of antioxidant enzymes in plants, such as ascorbate peroxidase (APX), superoxide dismutase (SOD), glutathione reductase (GR), catalase (CAT), peroxidase (POD), peroxiredoxin (PRX) and glutathione S-transferase (GST) participate in the detoxification of peroxides (Guo et al., 2023; Dixon and Edwards, 2010; Mittler, 2002).

PRXs constitute a class of thiol peroxidases characterised by redox−active cysteine or selenocysteine residues. PRXs are extensively involved in physiological and developmental processes in plant, including defence against pathogenic infection, mechanical wounding and diverse abiotic stresses. PRXs are classified into five distinct subfamilies, which include the 2-Cys PRX, 1-Cys PRX, PRX II, PRX Q, and glutathione peroxidase (GPX) according to the thiol−dependent enzymatic activity (Rouhier and Jacquot, 2005; Horta et al., 2010). GPX plays an important role in thiol-dependent redox regulation in plants. GPXs consist of multiple isoenzymes with distinct subcellular localisations which exhibit different tissue-specific expression patterns (Attacha et al., 2017). These isoenzymes participate in responses to environmental stress, ROS accumulation and immune defence, and they modulate root development and stomatal movement in plants (Miao et al., 2006). Tomato plants overexpressing a selenium-independent *GPX* maintain a significantly higher photosynthesis rates and fructose-1,6-bisphosphatase activity under chilling stress (Herbette et al., 2005).

Functional characterisation of GPX families highlights the essential role of *GPX* genes in maintaining cellular ROS homeostasis and stress adaption in plant (Zhang et al., 2024; Bela et al., 2025; do Carmo Santos et al., 2025). GPX efficiently catalyses the reduction of H_2_O_2_, organic peroxides, and phospholipid peroxides, thereby playing a critical role in stress defence and adaptive responses (Bela et al., 2015). The expression of *GhGPX* in yeast contributes to H_2_O_2_ scavenging in response to various stressors (Chen et al., 2017). Overexpression of the two wheat *GPXs* in *Arabidopsis* modulates transcript abundance of core regulatory factors involved in salt, H_2_O_2_, and abscisic acid (ABA) signalling pathways (*ABI1*, *ABI2*, *SOS1* and *RBOHD*), thereby promoting H_2_O_2_ tolerance (Zhai et al., 2013). In addition to maintaining low ROS content, GPX may function as a redox sensor, enabling the perception and response to changes in the plant redox state. *GPX* overexpression improves salt stress tolerance in rice (Diao et al., 2014), whereas knockout of *OsGPX3* severely impacts normal growth and development, inducing stress-related morphological changes through H_2_O_2_ accumulation (Passaia et al., 2013). Comprehensive studies on six GPXs have demonstrated that *GPX6* expression is upregulated when exposing *Lotus japonicus* to NaCl, cadmium, aluminium, and nitric oxide (Ramos et al., 2009). Thiol peroxidase GPX1 in osmotic stress response in rice, where it serves as a redox sensor and transducer (Zhou et al., 2022).

GPX is also associated with antioxidant enzymes and participates in the ROS scavenging. The downregulated expression of chloroplast GPX1 and GPX7 results in decreased activities of Cu/ZnSOD and MnSOD under high light intensity, coupled with excessive H_2_O_2_ accumulation (Chang et al., 2009; Asada, 1999; Foyer and Noctor, 2000). Furthermore, GPX acts as a signalling sensor through physically interacting with other proteins. *Arabidopsis* GPX3 may be involved in drought stress signal transduction and contribute to H_2_O_2_ scavenging in guard cells by interfering with the 2C-type serine/threonine protein phosphatases, ABI1 and ABI2, during ABA signalling reactions (Delaunay et al., 2002; Miao et al., 2006).

Although the roles of most antioxidant enzymes in plant growth and development have been extensively investigated, the functional mechanisms of GPXs in plants remain unclear. The presence of multiple GPX isoforms implies that these proteins fulfil vital biological functions. Early transcriptome analysis of the Jin A cytoplasmic male sterile (Jin A-CMS) line revealed that *GPX* was downregulated at the key stage of microspore abortion (Yang et al., 2014). Excessive ROS triggered DNA fragmentation and caused premature apoptosis of tapetum, and eventually resulted in microspore abortion in Jin A-CMS (Zhang et al., 2023, 2025). Given the role that GPX plays in enhancing plant tolerance to adverse environmental stresses, we conducted functional studies on *GPX* using several approaches, including genome-wide identification, subcellular localisation, gene silencing, and protein interaction analyses, with the aim of characterising the mechanism which GPX modulates ROS metabolism in the Jin A-CMS line.

## 2 Materials and methods

### 2.1 Plant materials and growth conditions

Jin A-CMS was selected from the offspring of interspecific hybrid bred ((*Gossypium hirsutum* × *G. thurberi*) × (*G. arboreum* × *G. hirsutum*)) in cotton (Yuan et al., 1996). The Jin A-CMS, maintainer Jin B (Jin B), and three-line hybrid F1 (F1) lines were grown at the Farm Station of Shanxi Agricultural University (37.42°N, 112.58°E). Wild-type *Arabidopsis*, *Nicotiana benthamiana*, and cotton were grown in a controlled growth chamber under specified conditions, referring to description of Zhang et al., 2025. Microspores were classified at the flowering stages (Zhang et al., 2023).

### 2.2 Genome-wide identification and functional analysis of glutathione peroxidase

Cotton genome sequences and annotation files were downloaded from CottonGen (https://www.cottongen.org/) (Chen et al., 2022; Yu et al., 2014). AtGPXs were used as reference sequences, then the whole protein sequences of three cotton species were scanned using the BLASTP program (e-value <1e^−5^) of TBtools (Chen et al., 2020; Rodriguez Milla et al., 2003). GPXs characteristics were analysed as previously described (Chen et al., 2022).

GPXs sequences were aligned using ClustalW program (Thompson et al., 1994). A maximum likelihood phylogenetic tree was constructed using the MEGA program (7.0) (Kumar et al., 2016) with 1,000 bootstraps and the Whelan-Goldman matrix (Whelan and Goldman, 2001), and was then drawn using the EvolView (He et al., 2016b).

The 2000 bp upstream region of translation initiation codon ATG of *GhGPX*s was selected and entered PlantCare (http://bioinformatics.psb.ugent.be/webtools/plantcare/html/) for promoter analysis (Lescot et al., 2002). The conserved motifs were analysed using MEME (http://meme.sdsc.e-du/meme/). The main parameters were set as follows: number of unique motifs, 10; maximum and minimum search widths, 50. All identified GhGPXs were verified to contain conserved domains using the NCBI CDD (http://www.ncbi.nlm.nih.gov/cdd) (Lu et al., 2020). The conserved motif, conserved domain, and gene structure were visualised using TBtools (Chen et al., 2020).

The gene background files of GO terms and KEGG pathways were derived from the cotton genetic improvement group of Hua Zhong Agricultural University (Wang et al., 2019). The GO and KEGG pathway enrichment analyses were performed using the Omicshare tools (https://www.omicshare.com/tools), taking false discovery rate (FDR)≤ 0.05 as a threshold.

The MCScanx and KaKs Calculator programs of TBtools were used to identify duplication events and the nonsynonymous mutation rate (Ka), synonymous mutation rate (Ks), and Ka/Ks values of homologous gene pairs that occurred in *GhGPXs*, respectively (Chen et al., 2020).

To further explore functions of *GhGPXs* in *G. hirsutum*, the RNA-seq data of and 4°C, 37°C, NaCl, PEG treatments, and different tissues (bract, petal, torus, stem, root, leaf, pistil, sepal and anther) of *G. hirsutum* (TM-1) (accession number: PRJNA490626) were downloaded from the NCBI (https://www.ncbi.nlm.nih.gov/) (Sayers et al., 2021).

### 2.3 RNA extraction and quantitative reverse transcription-polymerase chain reaction qRT-PCR

RNA was extracted from leaves and anthers (~0.1g) of Jin A-CMS, Jin B, and F1 using a Plant RNA Rapid Extraction Kit (Aidlab Biotechnologies Co. Ltd., Beijing, China). Reverse transcription and Real Time PCR were performed using PrimeScript™ RT Reagent Kit with gDNA Eraser and TB Green^®^ Premix Ex Taq™ II (Takara Biotech Co., Ltd., Dalian, China) at Bio-Rad CFX Connect fluorescent PCR amplifier (Bio-Rad, Hercules, CA, USA) according to the supplier’s instructions. *G. hirsutum EF1α* and *Arabidopsis Actin8* were used as the internal control genes for qRT-PCR. All primers were synthesised by Beijing Tsingke Biology Co., Ltd. (Beijing, China) (**Supplementary Table 1**). The relative gene expression level was calculated by the 2^-ΔΔCT^ method. Each experiment was conducted with three biological replicates, independently.

### 2.4 Plasmid construction and plant transformation

To construct recombinant plasmids, the target genes were cloned into the *Pro35S*:pRI101-AN, *Pro35S*:pCAMBIA1302, *Pro35S*:pTRV2, *Pro35S*:pET22b, PSYCE-35S, and PSYNE-35S plasmids by seamless cloning after plasmids were linearised using restriction enzymes (New England Biolabs, Beijing, China). Recombinant plasmids were constructed using ClonExpress II One Step Cloning Kit (Vazyme Biotechnology, Nanjing, China) according to the manufacturer’s instructions (Primers were shown in **Supplementary Table 1**). The recombinant *Pro35S*:pET22b plasmid was transformed into *Transetta (DE3)* Chemically Competent Cell (TransGen Biotech Co., Ltd., Beijing, China) according to the manufacturer’s instructions. Recombinant plasmids (*Pro35S*:pRI101-AN, *Pro35S*:pCAMBIA1302, *Pro35S*:pTRV2, PSYCE-35S, and PSYNE-35S) were transformed into *Agrobacterium tumefaciens* GV3101 (Shaanxi Breeding Biotech Co., Ltd., Yangling, China) according to the manufacturer’s instructions.

### 2.5 Subcellular localisation

Transformed bacteria of subcellular localisation were activated (28°C, 200 rpm, and OD_600_ to 1.0) and collected. Bacteria cultures were resuspended in a resuspension solution containing 10 mM magnesium chloride, 120 μM acetosyringone, and 10 mM 2-Morpholinoethanesulfonic acid Healthy and robust tobacco plants (4–5 weeks old) were selected for *Agrobacterium*-mediated transient transformation. The green fluorescent protein (GFP) signal was observed using laser confocal microscopy (Leica DMi8, Wetzlar, Germany) in 2 d after injection, with an excitation wavelength of 488 nm and an emission wavelength of 507 nm. The fluorescence excitation and emission wavelengths of chloroplast auto-fluorescence were 633nm and 685 nm, respectively.

### 2.6 Metabolite and enzyme measurements

Samples of fresh tissue materials were accurately weighed (~0.1g) and extracted at room temperature with extraction solution. Samples were extracted in 10 mM Tris-HCl (pH 7.6) with 20 mM iodoacetic acid and pre-incubated at 25 °C for 30 min for GPX activity assay. Reaction assay of GPX activity contained 1 mM ethylenediaminetetraacetic acid, 200 mM NADPH, 1 mM NADPH-dependent thioredoxin reductase, 2 mM TRX, and 250 mM H_2_O_2_. Reaction was followed through NADPH oxidation at 340 nm (Müller and Winter, 2017; Castella et al., 2017). GST activity was determined by the change in substrate concentration in reaction, which 1 mM GSH conjugates with the 1 mM 1-chloro-2,4-dinitrophenyl (He et al., 2016a). The formation of the reaction product was monitored at 340 nm; one unit of enzymatic activity was defined as the production of 1 μmol of product. Dehydroascorbate reductase (DHAR) activity was determined by an increase in absorbance at 295 nm due to dehydroascorbate reduction (Knorzer et al., 1996). Each experiment was conducted with three biological replicates, independently.

### 2.7 VIGS assays and overexpression *Arabidopsis* assays

We constructed recombined tobacco rattle virus (TRV)-based pTRV2 plasmid (Pang et al., 2013). Leaves of Jin B were utilised for injection after the cotyledons had fully unfolded. *TRV:00* was served as controls, qRT-PCR was used for verifying silenced gene efficiency. Bacteria cultures harbouring the pRI101-AN recombined plasmid were used to transform wild-type *Arabidopsis* plants via the floral-dip method (Bent, 2006). *Agrobacterium* cultures were resuspended in Murashige and Skoog (MS) medium (5% sucrose, 0.02% Silwet L-77). The homozygous strains were screened on MS solid medium (3% sucrose). qRT-PCR was used for verifying overexpressing gene efficiency. Anther developmental periods were classified as previously described for *Arabidopsis* (Sanders et al., 1999).

### 2.8 ROS detection

The Nitroblue tetrazolium chloride (NBT) and 3,3’-diaminobenzidine (DAB) staining were performed according to a published method (Wu et al., 2015). Samples were soaked in the staining solution (0.2 mM NBT) for 2 h and then decolorised with 95% ethanol. The samples were soaked in the staining solution (1 mg/mL DAB) for 24 h and then decolorised with acetic acid, glycerin, and 95% ethanol (1:1:3). The samples were observed using a stereomicroscope (Olympus SZX16, Germany). ^1^O_2_ was measured using Singlet Oxygen Sensor Green reagent (Beijing Biolab Technology Co., Ltd., Beijing, China) according to the manufacturer’s instructions. The samples were observed using laser confocal microscopy (Leica DMi8, Wetzlar, Germany).

### 2.9 Quantitative ROS assays and malondialdehyde content determination

Quantitative ROS and MDA content assays were measured in silenced cotton and overexpressing *Arabidopsis.* H_2_O_2_ was measured by monitoring the 415 nm of the titanium-peroxide complex following the method described by Liu et al. (2007). For measuring O_2_^−•^ levels, the reaction system contained 50 mM phosphate buffer (pH 7.8) supplemented with 17 mM sulphanilic acid, 10 mM hydroxylamine hydrochloride, and 7 mM 1-naphthylamine, with absorbance determined at 530 nm (Liu et al., 2007). For MDA quantification, leaf samples were homogenized in 20% trichloroacetic acid (w/v), followed by centrifugation. The collected supernatant was mixed with 0.5% thiobarbituric acid reagent, boiled for 30 min. Absorbance values of the reaction solution were recorded spectrophotometrically at 450 nm, 532 nm and 600 nm for subsequent calculation of MDA content (Farhadi and Ghassemi-Golezani, 2020). Determination of ^1^O_2_ was performed by Plant Singlet Oxygen assay kit (GENMED, UAS) to measure dimethyl-4-nitrosoaniline reduction of peak absorption under 440 nm, according to the manufacturer’s instructions. Each experiment was conducted with three biological replicates, independently.

### 2.10 ROS-scavenging enzyme activities assays

Pre-cooling 50 mM phosphate buffer (pH 7.8) was used for extract solution of enzyme activities. Determination of SOD, POD, and CAT activities were performed as described previously (Demircan et al., 2020). Each experiment was conducted with three biological replicates, independently.

### 2.11 Recombinant proteins expression, purification and *in vitro* pull-down assays

Recombinant expression plasmids were transferred to Transetta DE3 chemically competent cells (Beijing TransGen Biotech Co., Ltd., Beijing, China). A 13% Sodium dodecyl sulphate-polyacrylamide gel electrophoresis (SDS-PAGE) gel was made as Zhang et al. (2025) described. Recombinant protein was purified using ÄKTA™ Pure (Cytiva, Marlborough, Massachusetts, USA) according to the manufacturer’s instructions. Total protein in the buds was extracted using Western and IP Cell lysis buffer (Shanghai Beyotime Biotechnology Co., Ltd., Shanghai, China) according to the manufacturer’s instructions. Pull-down assays were performed as described previously (Louche et al., 2017).

### 2.12 Bimolecular fluorescence complementation assay

Recombinant plasmids with yellow fluorescent protein C-terminal and N-termini were expressed in *Agrobacterium tumefaciens* GV3101 cells, and bacteria cultures were mixed 1:1 by volume and injected into the lower epidermis of tobacco leaves. The signal was observed using laser confocal microscopy in 2 d after injection.

### 2.13 Yeast two-hybrid complementation assay

Recombinant plasmids, including pGADT7 and pGBKT7, were co-transformed into yeast (Lin and Lai, 2017). Three 10-fold dilutions were prepared under sterile conditions. SD/-Trp/-Leu, SD/-Trp/-Leu/-His, and SD/-Trp/-Leu/-His/-Ade media were used for testing yeast growth.

### 2.14 Interaction protein binding-site analysis

AlphaFold 2 software was used to model three-dimensional structures of interaction protein (Yang et al., 2023). Subsequently, we used the HDOCK software (http://hdock.phys.hust.edu.cn/) (Yan et al., 2017) for protein-protein docking, yielding a variety of complex structures. These structures were meticulously sorted and screened based on their respective confidence scores. The complex structure with the highest confidence score was identified as the target for analysis. We then employed PyMOL software (Schrödinger, LLC., NY, USA) to delve into the binding site of the protein-protein complex.

### 2.15 Statistical analysis

Three separate experiments or biological replicates were analysed using IBM SPSS 25.0 (IBM Corp., NY, USA), using a one-way ANOVA followed by Duncan’s multiple comparison test. Charts were drawn using GraphPad Prism 8.0 (GraphPad Software Inc., La Jolla, CA, USA).

## 3 Results

### 3.1 Genome-wide identification and characterisation of glutathione peroxidase

Eight GPXs in *Arabidopsis* were used for database mining using the BLAST search tool (Rodriguez Milla et al., 2003). A total of 16, 8, and 8 GPXs were identified in cotton species, *G. hirsutum*, *G. arboreum*, and *G. raimondii*, respectively. These GPXs were systematically renamed based on their chromosomal positions. Detailed information of *GPXs* and their corresponding proteins was shown in **Supplementary Table 2**. The open reading frames ranged from 384 to 729 bp in length and encoding proteins ranged from 127 to 242 amino acids. The predicted molecular weights (MW) and theoretical isoelectric points varied from 14.33 to 27.46 kDa and 4.33 to 9.73, respectively. All members of the GPX family were predicted to be hydrophilic protein (**Supplementary Table 2**). The hydrophilic nature of GPXs facilitates sufficient binding to soluble H_2_O_2_ and its core antioxidant function against oxidative stress. Genome-wide identification of the cotton *GPX* gene family yielded inconsistent member lists between the present study and the previous report of Chen et al. (2017) (**Supplementary Table 3**). These differences were primarily attributed to the substantial improvement in the completeness and annotation quality of the latest cotton genome assembly.

**Figure 1:**
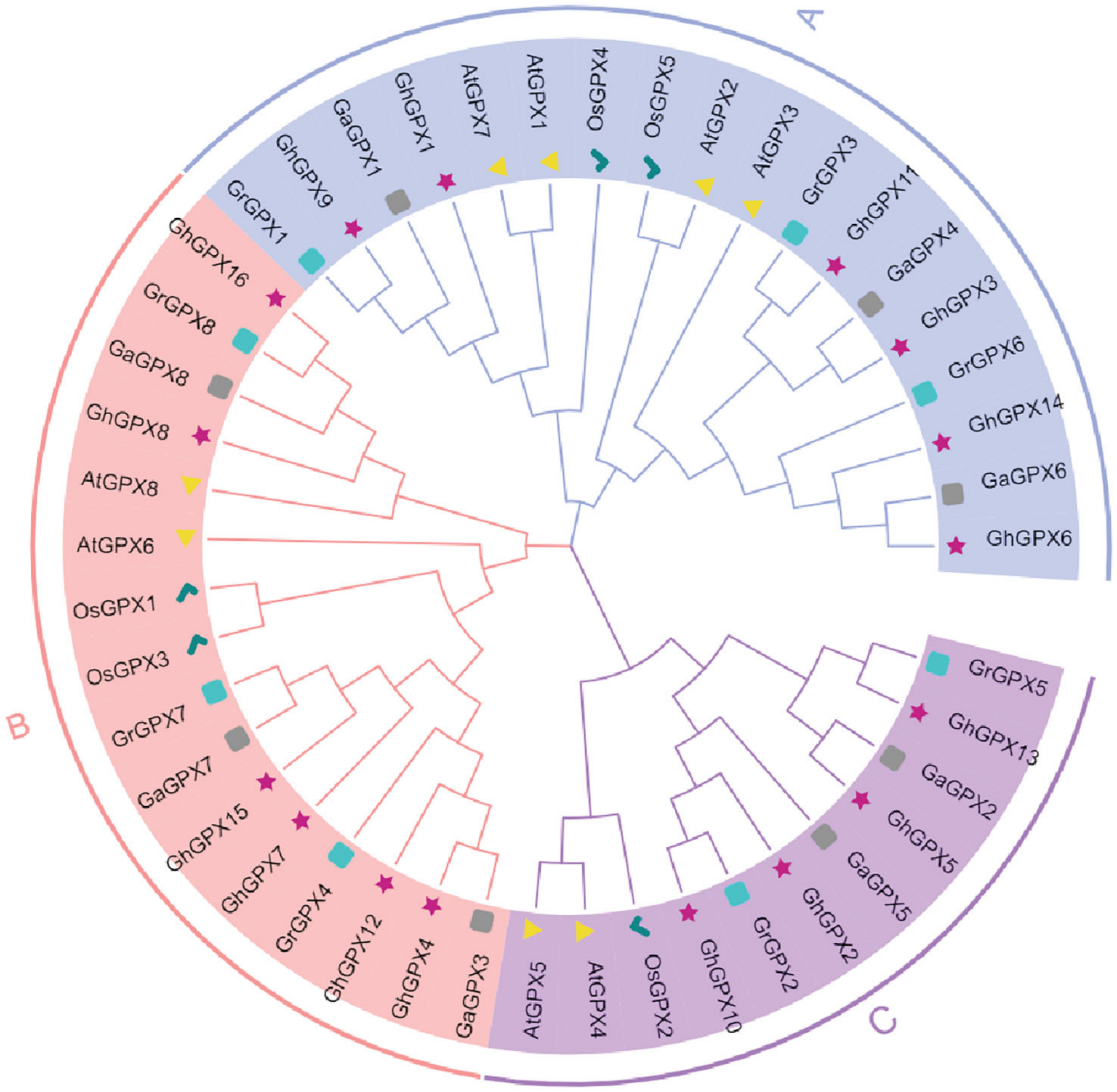
Phylogenetic analysis of GPXs from *G. hirsutum, G. arboreum, G. raimondii*, *Arabidopsis*, and *rice*. Based on the full-length protein sequences, the phylogenetic tree was constructed using the maximum likelihood method. Distinct coloured numbers represent different subgroups **(A–C)**.

To investigate the evolutionary relationships among GPXs, a phylogenetic tree was constructed using MEGA 7.0 based on GPX sequences derived from *G. hirsutum*, *G. arboreum*, *G. raimondii*, *Arabidopsis*, and rice (**Figure 1**). The GPXs was classified into three subgroups (A–C), with at least 1000 bootstraps supported on phylogenetic trees. All species belonged to three subgroups, suggesting that GPXs are evolutionarily conserved across different species.

**Figure 2:**
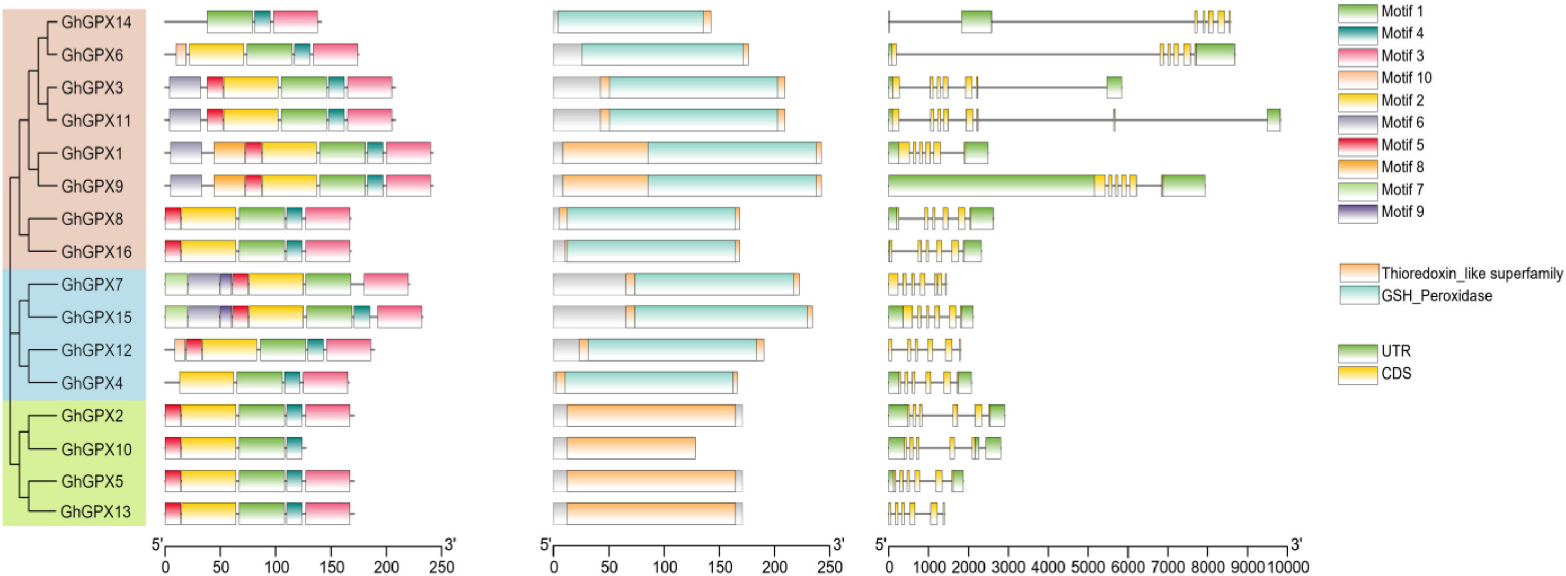
Phylogenetic tree, conserved motif, conserved domain, gene structure analysis of GhGPXs in *G. hirsutum*. Introns and exons are represented by thin lines and yellow boxes, respectively. The UTR is shown in a green box.

The phylogenetic analysis of GhGPXs revealed that they were grouped into three subgroups (**Figure 2**). Using the MEME program, 10 conserved motifs were identified in the GhGPXs, and all GhGPXs contained the “Thioredoxin_like superfamily” domain. Except for GhGPX2, GhGPX5, GhGPX10, and GhGPX13, all other GhGPXs contained the “GSH_Peroxidase” domain. Additionally, with the exception of *GhGPX3, GhGPX7*, *GhGPX10*, *GhGPX11* and *GhGPX14*, most *GhGPXs* consisted of six exons and five introns, indicating structural conservation during evolution.

**Figure 3:**
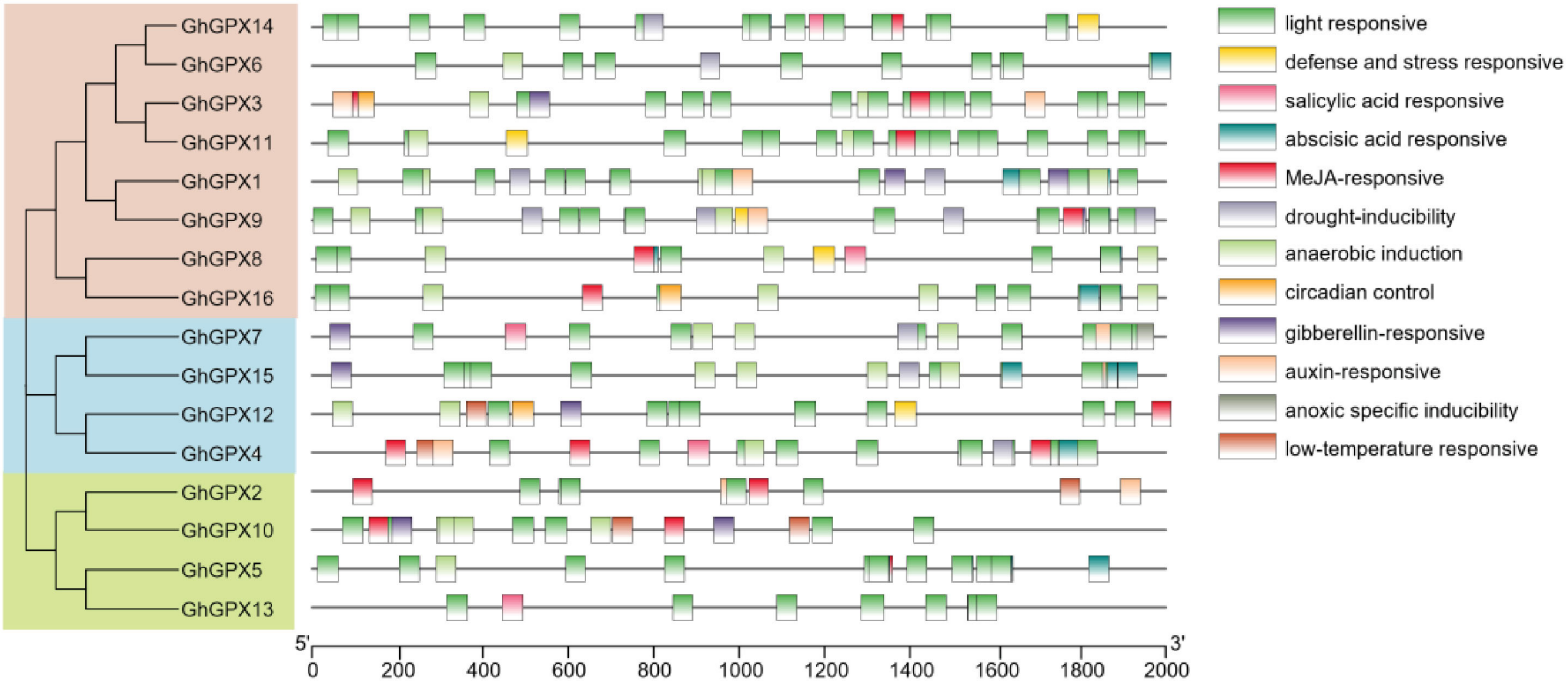
Cis-acting element analysis of *GhGPX*s promotors in *G. hirsutum*.

**Figure 4:**
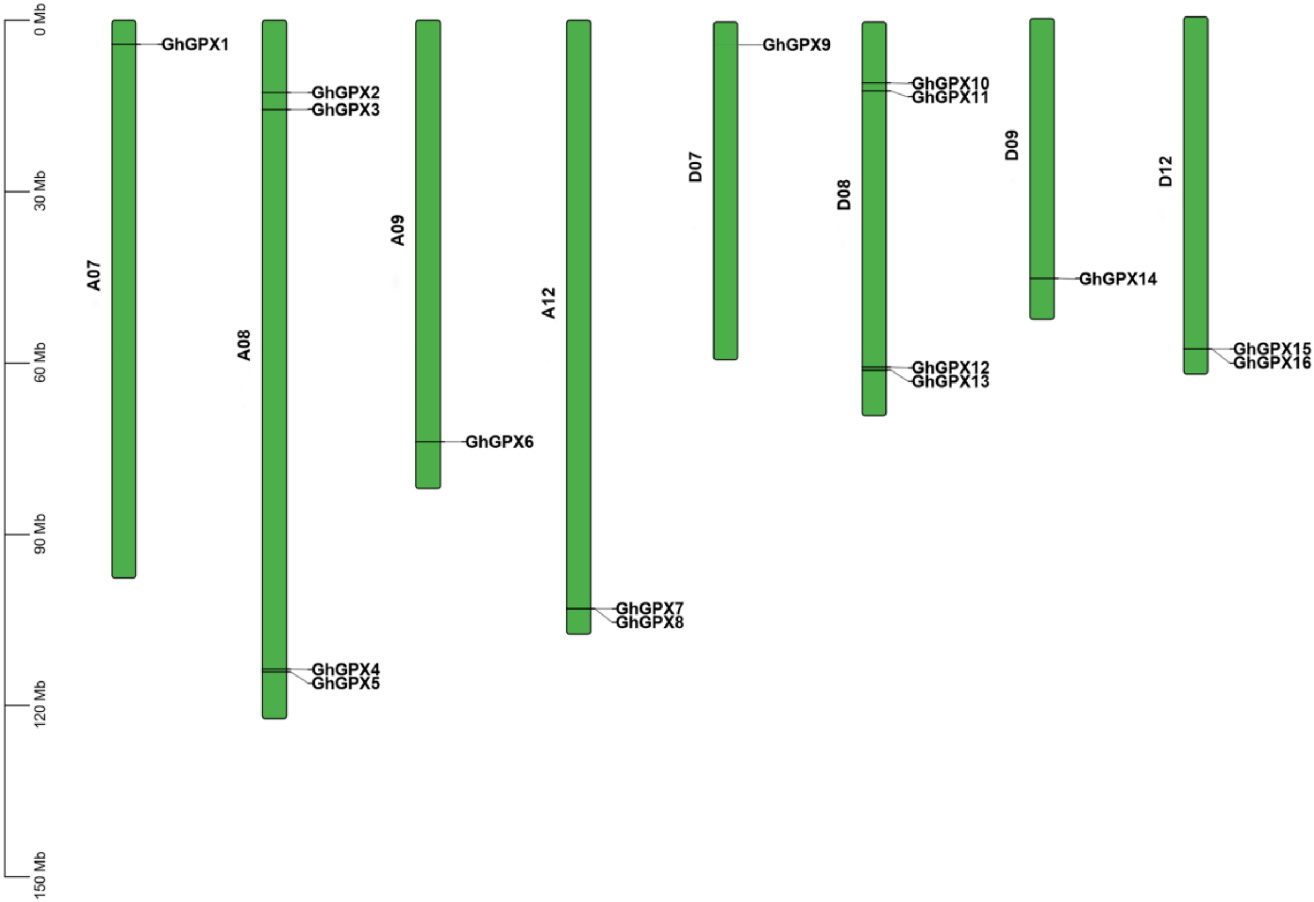
The chromosomal localisation of *GhGPXs* in *G. hirsutum*.

Promoter cis-elements are crucial for regulating gene expression. A total of 34 stress-responsive cis-acting elements were identified in the promoter regions of *GhGPXs*, including light responsiveness, drought stress, plant hormone signalling, and cold stress response elements (**Supplementary Table 4**). These elements were classified into 12 categories and presented in **Figure 3**. These findings suggest that *GhGPX* expression is regulated by plant hormones, defence signalling pathways, and abiotic stress conditions during cotton growth and development. Chromosomal localisation analysis revealed that *GhGPXs* were distributed across 8 chromosomes (**Figure 4**).

**Figure 5:**
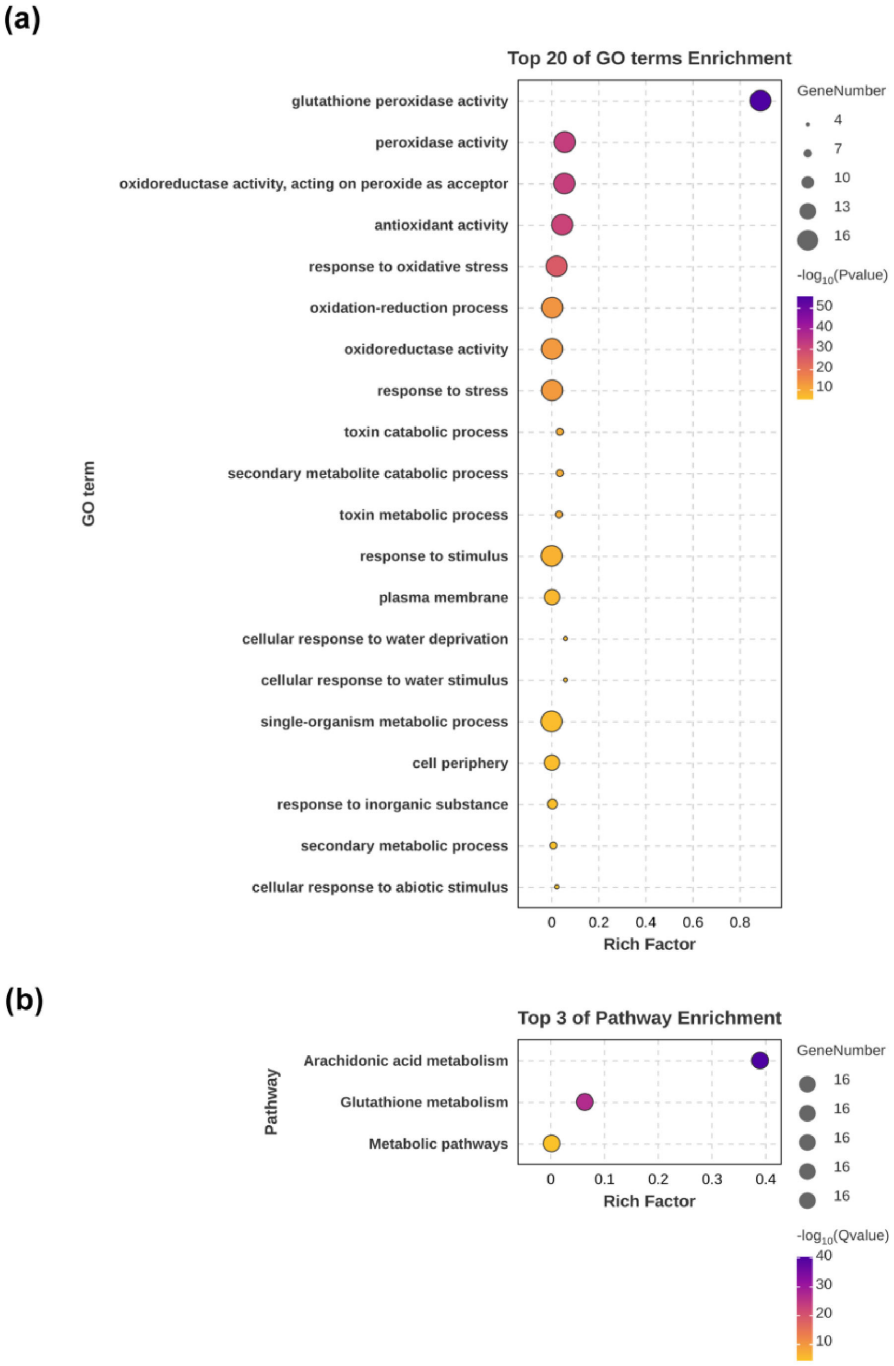
The analysis of GO **(a)** and KEGG **(b)** enrichment of *GhGPXs* in *G. hirsutum*. Rich Factor indicates the ratio of genes number located in GO term to the total genes number located in GO term in all background genes. GeneNumber indicates the number of genes located in GO term.

To further explore the functional roles of *GhGPXs* in signalling pathways and biological processes, we performed Gene Ontology (GO) and Kyoto Encyclopedia of Genes and Genomes (KEGG) pathway enrichment analyses of *GhGPX* family. A total of 111 GO terms were significantly enriched (**Supplementary Table 5**). The enriched biological process terms included cellular redox homeostasis and redox reactions. Molecular function analysis revealed significant enrichment of GhGPXs, redox enzyme, and antioxidant activities. Cellular component annotations indicated that *GhGPXs* were predominantly localised in plastids and plasma membranes. The top 20 significantly enriched GO terms were visualised using the OmicShare tool (**Figure 5a**). GO enrichment were largely associated with redox process and stress response, and the encoded proteins possessed typical antioxidant enzyme activities. KEGG pathway enrichment analysis revealed that arachidonic acid metabolism and glutathione metabolism were enriched (**Figure 5b**; **Supplementary Table 6**). These results indicated the redox function of *GhGPXs* (Zhou et al., 2022).

**Figure 6:**
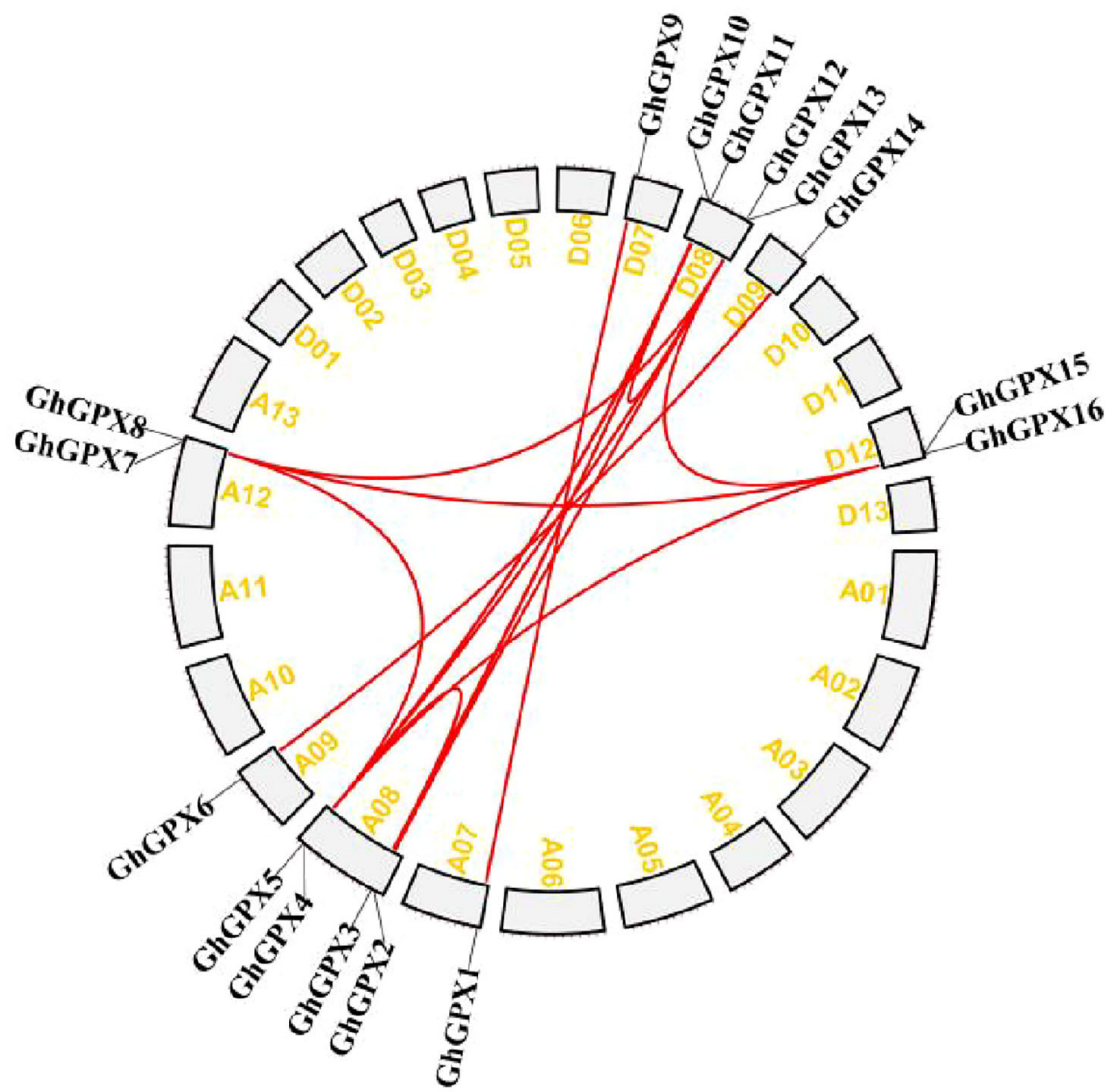
Collinearity analysis of *GhGPXs* in *G. hirsutum*.

Seventeen pairs of paralogous genes were identified in *G. hirsutum*, using MCScanX. Collinearity analysis demonstrated that segmental duplication is the primary driving force for *GhGPX* family expansion, whereas tandem duplication rarely occurred (**Figure 6**). Most duplicated paralog pairs exhibited Ka/Ks<1, indicating dominant purifying selection constrained drastic functional alteration during evolution (**Supplementary Table 7**). Two gene pairs lacked calculable Ks values, suggesting their duplication occurred at an early evolutionary stage. Minor sequence variation among duplicated genes might trigger functional divergence in ROS regulation.

**Figure 7:**
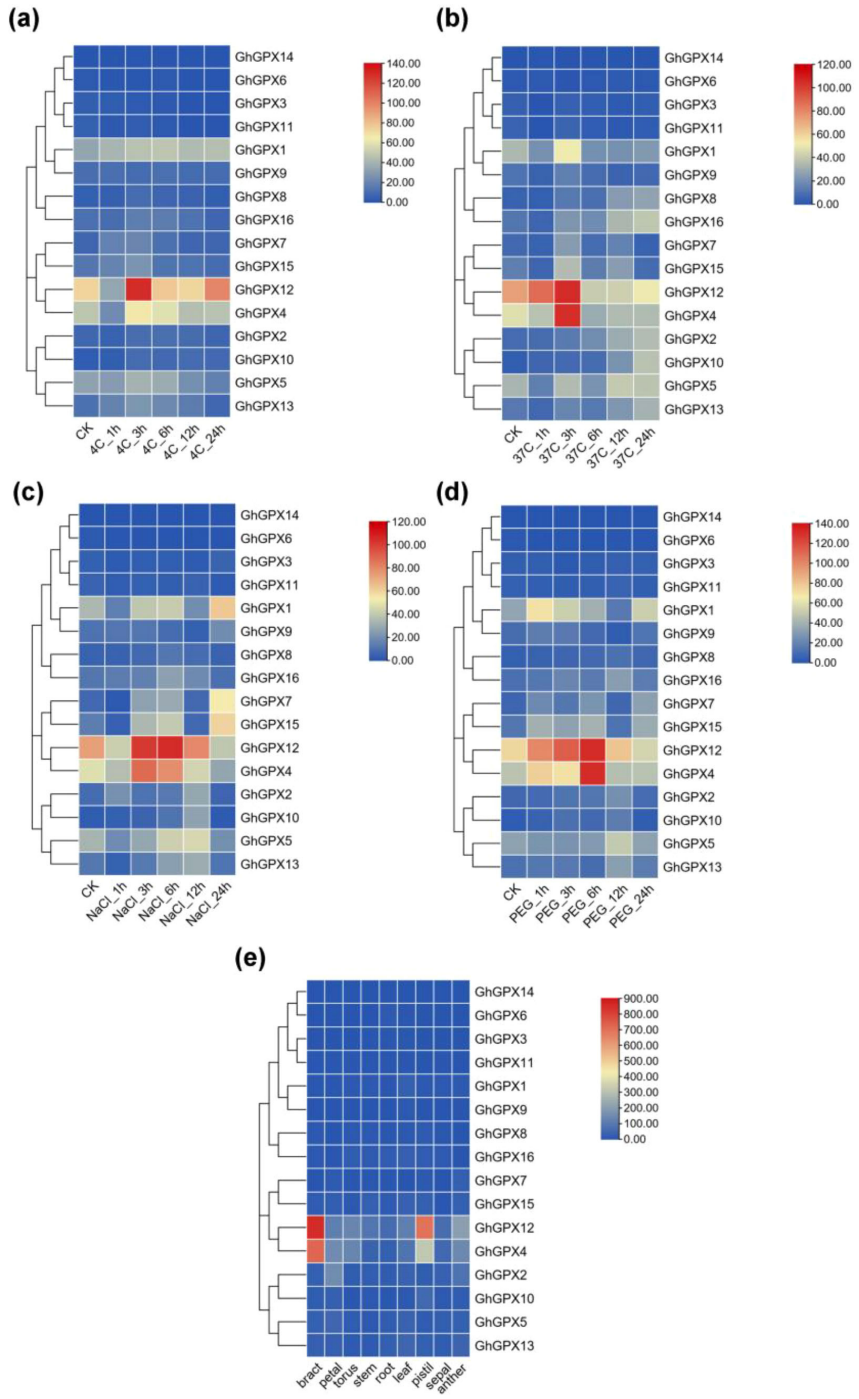
Expression profile analysis of *GhGPXs* in 4°C **(a)**, 37°C **(b)**, NaCl **(c)**, PEG treatments **(d)**, and different tissues **(e)**.

FPKM values derived from the transcriptome data were used to analyse the expression patterns of *GhGPXs* across different tissues and under different stress conditions (**Figure 7**). Following exposure to low-temperature, high-temperature, NaCl and PEG stress for 3 h, the expression of *GhGPX4* and *GhGPX12* were upregulated (**Figures 7a–d**), suggesting that these genes play key roles in stress responses in cotton. Expression of the other *GhGPXs* did not significantly change under stress conditions. Tissue-specific expression analysis revealed that *GhGPX4* and *GhGPX12* were highly expressed in the bracts, and *GhGPX12* notably upregulated in the pistil (**Figure 7e**).

### 3.2 Altered glutathione peroxidase gene expression levels and enzyme activity

**Figure 8:**
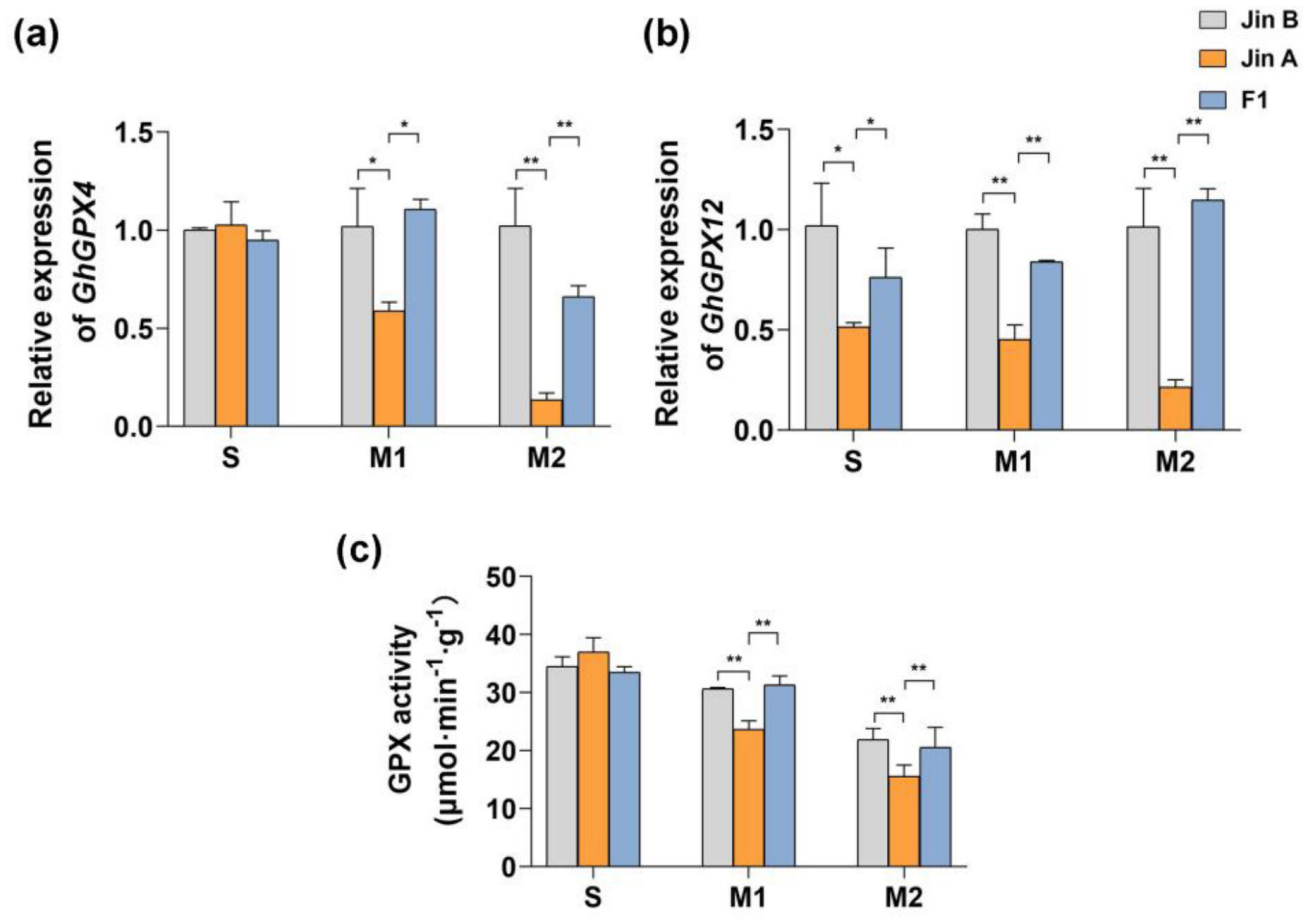
The assay of *GhGPX4* **(a)**, *GhGPX12* **(b)** gene expression and GPX enzyme activity **(c)** in Jin A-CMS, its maintainer Jin B and three-line hybrid F1. S (before the microspore abortion stage), M1 (the microspore abortion stage) and M2 (after the microspore abortion stage). The data are means ± SD of three biological replicates. *P<0.05; **P<0.01 according to one-way ANOVA (Duncan’s multiple comparison test).

We determined the expression levels of *GhGPX4* and *GhGPX12*, as well as GPX enzyme activity, at different developmental stages in anthers from the Jin A-CMS, Jin B, and F1 lines. The results demonstrated that compared with the maintainer line, the expression levels of *GhGPX4* and *GhGPX12* were significantly downregulated in the Jin A-CMS line at and after the microspore abortion stage, accompanied by a decrease in GPX activity. In contrast, in the F1, the expression levels of *GhGPX4* and *GhGPX12* were upregulated, and GPX activity correspondingly increased, showing no statistically significant differences compared with those in Jin B (**Figures 8a–c**). These findings suggest that *GhGPX4 and GhGPX12* play important roles in microspore development in the Jin A-CMS line.

### 3.3 Subcellular localisation of glutathione peroxidase genes encoded protein

**Figure 9:**
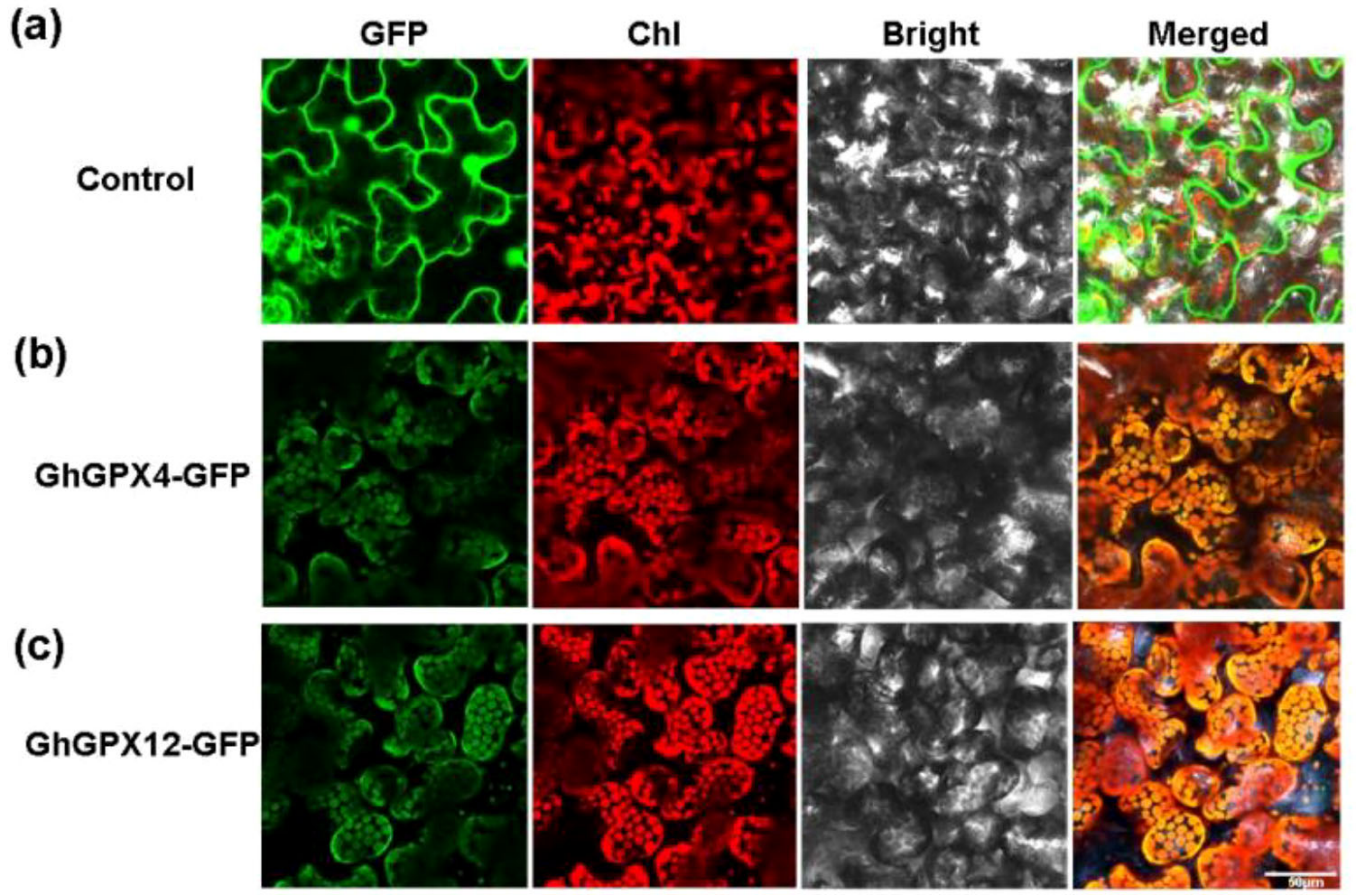
Subcellular localisation of *GhGPX4* and *GhGPX12* genes encoded protein. Confocal images represent control protein **(a)**, GhGPX4-GFP **(b)**, and GhGPX12-GFP **(c)** fusion protein expression, respectively. GhGPX4 and GhGPX12 were located in chloroplasts in *N. benthamiana*. GFP was excited at 488 nm and imaged simultaneously with chlorophyll auto-fluorescence excited at 633nm. GFP (green fluorescence), RFP (chloroplast auto-fluorescence), Bright (bright field), Merged (merged field), bar = 50 μm.

We used a homologous cloning approach and designed gene-specific primers to amplify the full-length coding sequences (CDS) of *GhGPX4* and *GhGPX12* from the Jin A-CMS line. Sequence analysis revealed that the CDS of *GhGPX4* and *GhGPX12* were 501 and 570 bp in length (**Supplementary Figure 1**), respectively. To confirm subcellular localisation of encoded proteins, we selected *N. benthamiana* as a model system and performed transient expression assays for both candidate genes (**Figure 9**). The results demonstrated that GhGPX4 and GhGPX12 were mainly located in chloroplasts.

### 3.4 Role of glutathione peroxidase in ROS metabolism

**Figure 10:**
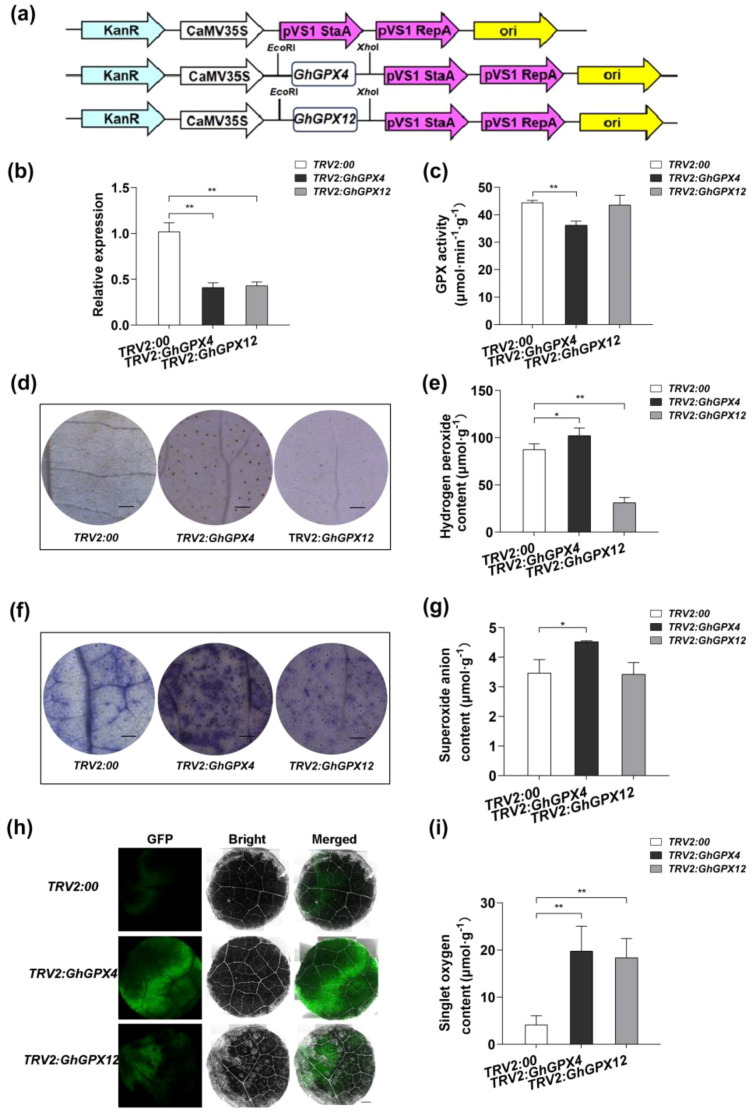
ROS detection of *GhGPX*s silencing cotton. **(a)** Recombinant plasmids construction of *GhGPX*s silencing cotton. **(b)** Relative expression of *GhGPXs*. **(c)** The activity of GPX. H_2_O_2_ staining **(d)** and content **(e)** of *GhGPXs* silencing cotton in leaves, bar =1 mm. O_2_^^-^•^ staining **(f)** and content **(g)** of *GhGPXs* silencing cotton in leaves, bar =1 mm. ^1^O_2_ staining **(h)** and content **(i)** of *GhGPXs* silencing cotton in leaves, bar =1 mm. *TRV2: 00* (pTRV2, negative control), *TRV2: GhGPX4* (pTRV2-*GhGPX4*), *TRV2: GhGPX12* (pTRV2-*GhGPX12*). The data are means ± SD of three biological replicates. *P< 0.05; **P<0.01 according to one-way ANOVA (Duncan’s multiple comparison test).

We constructed *GhGPXs* silencing plasmids and transformed them into *Agrobacterium tumefaciens* GV3101 cells (**Figure 10a**). Plants transformed with an empty pTRV2 plasmid were used as negative controls. The expression levels of *GhGPX4* and *GhGPX12* in the gene-silenced cotton were reduced to 40.26% and 42.03% of those in the control, respectively (**Figure 10b**). GPX activity was reduced in *GhGPX4*-silenced cotton (**Figure 10c**). DAB and NBT staining were performed on gene-silenced cotton leaves to assess the accumulation of H_2_O_2_ and O_2_^^-^•^. H_2_O_2_ and O_2_^^-^•^ contents were significantly elevated in the *GhGPX4*-silenced cotton compared with those in the control (**Figures 10d, f**). Quantitative measurement of H_2_O_2_ and O_2_^^-^•^ showed similar results (**Figures 10e, g**). Additionally, results of ^1^O_2_ detection and quantitative determination showed higher ^1^O_2_ accumulation in leaves of *GhGPX4*- and *GhGPX12*-silenced cotton compared with those in the control (**Figures 10h, i**).

To further explore the functions of *GhGPXs*, we constructed pRI101-*GhGPX4* and pRI101-*GhGPX12* overexpressing plasmids and obtained a homozygous generation of transgenic *Arabidopsis* (**Supplementary Figure 2**). Recombinant plasmid maps were shown in **Supplementary Figure 3a**. The qRT-PCR assay was performed on the *Arabidopsis* lines, whereafter those with high relative gene expression levels were selected for further analysis (**Supplementary Figure 3b**). GPX activity increased in *GhGPX4*-overexpressed plants (**Supplementary Figure 3c**). DAB staining showed that in *GhGPX4*-overexpressed plants, pollen staining was not significantly different from that in wild type at stages 8 and 9 but was markedly reduced at stages 6, 7, and 11, suggesting decreased H_2_O_2_ accumulation (**Supplementary Figure 3d**). NBT staining of pollen from the *GhGPX4*- and *GhGPX12*-overexpressed *Arabidopsis* plants was used for determining O_2_^^-^•^ levels (**Supplementary Figure 3e**). Compared with the wild type, *GhGPX4*-overexpressed *Arabidopsis* exhibited reduced staining at pollen developmental stages 6, 7, and 9–11, indicating a lower accumulation of O_2_^^-^•^ in the pollen. In contrast, no significant difference in staining was observed between the *GhGPX12*-overexpressed plants compared and wild-type plants. Quantitative ROS assays of leaves revealed reduced levels of H_2_O_2_, O_2_^^-^•^ and ^1^O_2_ content in *GhGPX4*-overexpressed plants, while only O_2_^^-^•^ content decreased in *GhGPX12*-overexpressed plants compared with wild-type controls (**Supplementary Figure 4**). These results suggest that *GhGPX4* plays a key role in ROS scavenging. To investigate the functional divergence of GhGPX4 and GhGPX12 in ROS scavenging, we conducted a comparative analysis of their sequence alignments and secondary structural feature. C-terminal divergent segment between GhGPX4 and GhGPX12 triggers global secondary structure remodelling with concurrent increases in α-helix, β-sheet, and random coil, which might impact the molecular interactions, ultimately leading to difference of function (**Supplementary Figures 5**, **6**) (Shi et al., 2021; Chen et al., 2025).

### 3.5 Screening and validation of glutathione peroxidase interacting proteins

**Figure 11:**
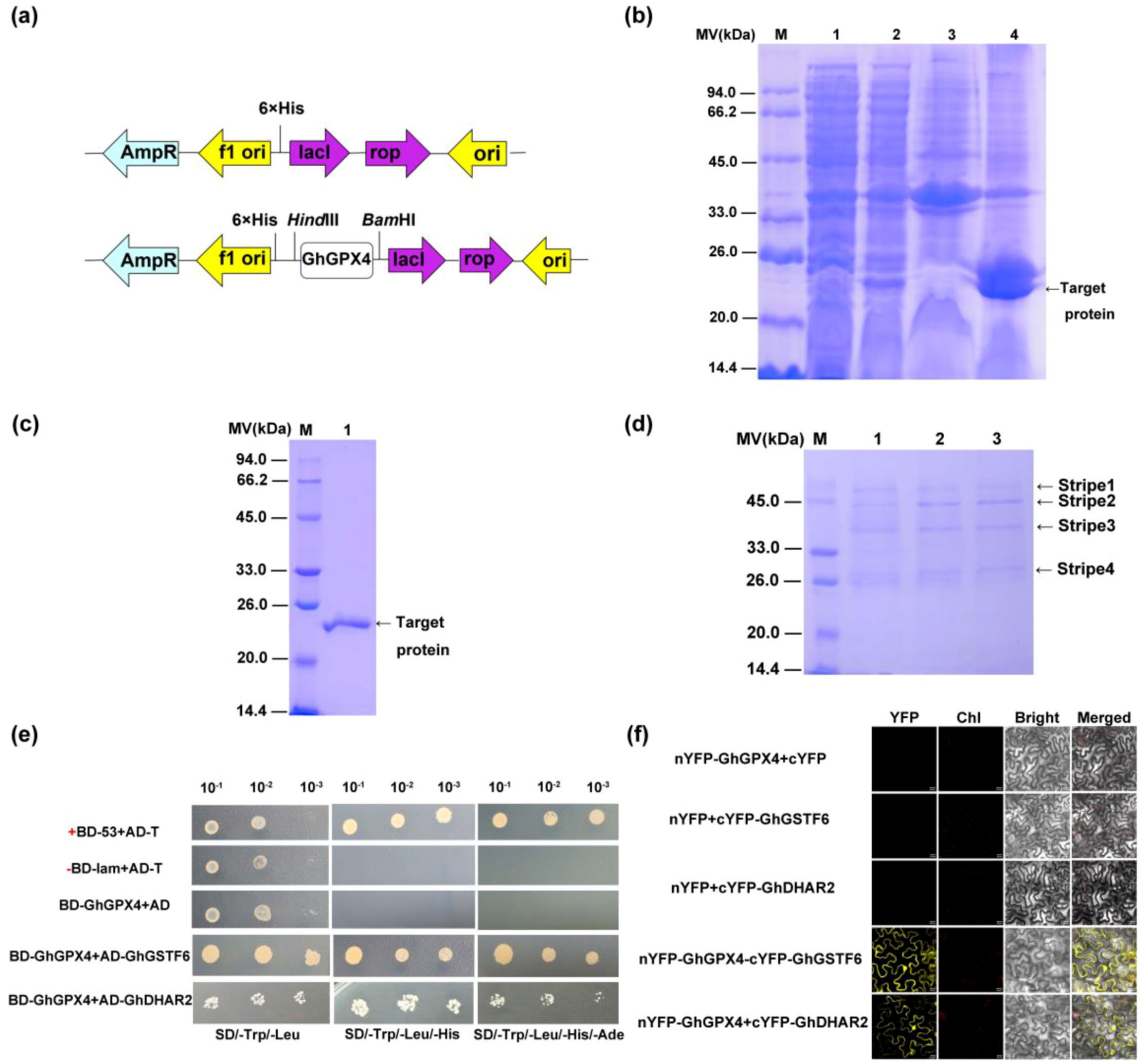
Screening and validation of GhGPX4 interacting proteins. **(a)** Construction of GhGPX4-His recombinant protein. **(b)** Expression of the GhGPX4-His recombinant protein in *E*.*coli*. M, marker; 1, control supernatant; 2, GhGPX4-His recombinant protein supernatant; 3, control sediment; 4, GhGPX4-His recombinant protein sediment. **(c)** The purified recombinant protein exhibited a single band between 20–26 kDa on SDS−PAGE gel. M, marker; 1, GhGPX4. **(d)** GhGPX4 interacting protein screening. M, marker; 1~3, potential interacting proteins with GhGPX4. Interaction verification of GhGPX4 and GhGSTF6, GhDHAR2 by yeast two hybrid system **(e)** and BiFC **(f)**. YFP (yellow fluorescence), Chl (chloroplast autofluorescence), Bright (bright field), Merged (merged field), bar = 20 μm.

Expression profiling and functional analysis of *GhGPX* indicated that *GhGPX4* played a crucial role in ROS metabolism in the Jin A-CMS line. Therefore, *GhGPX4* was selected as the candidate gene, and a recombinant protein constructed to investigate its involvement in the ROS metabolic pathway (**Figure 11a**). The MW of the GhGPX4-PET22b recombinant protein was in the range of 20–26 kDa (**Figure 11b**), which is consistent with the expected size and suitable for use in downstream experiments.

SDS-PAGE showed a single specific band of the purified recombinant GhGPX4 protein between 20–26 kDa, matching its predicted molecular mass (**Figure 11c**). This result aligned with experimental expectations and confirmed the suitability of the protein for further analysis. Mass spectrometry was performed on the purified protein band (**Supplementary Figure 7**), revealing a sequence coverage of 61% for the GhGPX4 recombinant protein, with 27 matching peptide segments and high sequence similarity. These results confirmed that the purified protein was GhGPX4.

His pull-down assays identified four protein bands interacting with GhGPX4 within an MW range of 26–66.2 kDa (**Figure 11d**). The distinct bands were excised for mass spectrometry analysis, and six potential interacting proteins were identified: V-type proton ATPase subunit E, glyceraldehyde-3-phosphate dehydrogenase 2, L-APX 2, two GSTs (GhGSTF6 and GhDHAR2), and monodehydroascorbate reductase 5 (**Supplementary Table 8**). To validate the interactions, the GLA4 yeast two-hybrid system was employed. Self-activation assays showed that no autonomous activation in the GhGPX4-pGBKT7 recombinant plasmid, indicating its suitability for downstream interaction studies (**Supplementary Figure 8**). Using yeast two-hybrid, two interacting proteins were identified, including GhGSTF6 and GhDHAR2 (**Figure 11e**). The remaining four proteins showed no evidence of interaction by yeast two-hybrid. And BiFC assays were used to verify the interactions. In contrast to the subcellular localisation of GhGPX4, the interaction occurred in the nucleus and cytoplasm (**Figure 11f**). These results may be attributable to explain the dynamic intracellular behaviour of GhGPX4 and its diverse functional roles.

We performed molecular docking analysis of the interacting proteins and their corresponding binding sites. The molecular docking confidence score of GhGPX4 and GhGSTF6 was 0.8951 (**Supplementary Figure 9a**), and that of GhGPX4 and GhDHAR2 was 0.7111 (**Supplementary Figure 10a**). These two protein groups exhibited mutual interactions with each other. The interaction mode primarily involved the formation of hydrogen bonds and salt bridges (**Supplementary Figures 9b**, **10b**).

### 3.6 The altered glutathione S-transferase activity in Jin A-CMS

**Figure 12:**
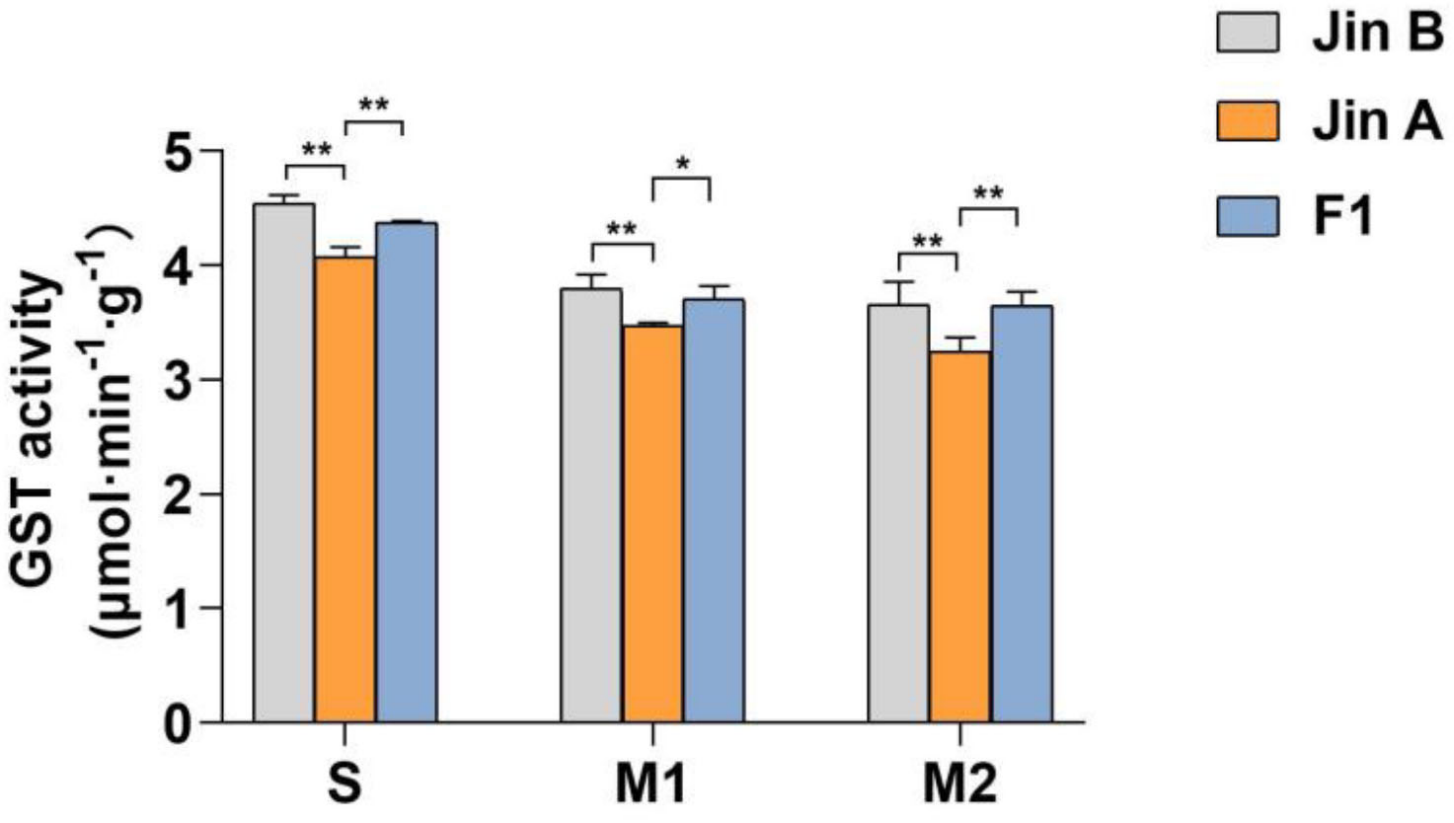
The activity of GST enzyme in Jin A-CMS, its maintainer line Jin B, and three-line hybrid F1. S (before the microspore abortion stage), M1 (the microspore abortion stage) and M2 (after the microspore abortion stage). The data are means ± SD of three biological replicates. *P<0.05; **P<0.01 according to one-way ANOVA (Duncan’s multiple comparison test).

Compared with the maintainer line, the Jin A-CMS line exhibited reduced GST (**Figure 12**) and DHAR activity (Zhang et al., 2025) at the key stage of microspore abortion. This result was consistent with our prior experiment regarding redox homeostasis alterations in the Jin A-CMS line. Changes in intracellular redox homeostasis and abundance of antioxidant enzymes during microspore development influenced cellular ROS levels, which were closely associated with male sterility phenotypes (Zhang et al., 2023).

### 3.7 Changes of antioxidant enzymes in *GhGPX4* silenced cotton and overexpressed *Arabidopsis*

**Figure 13:**
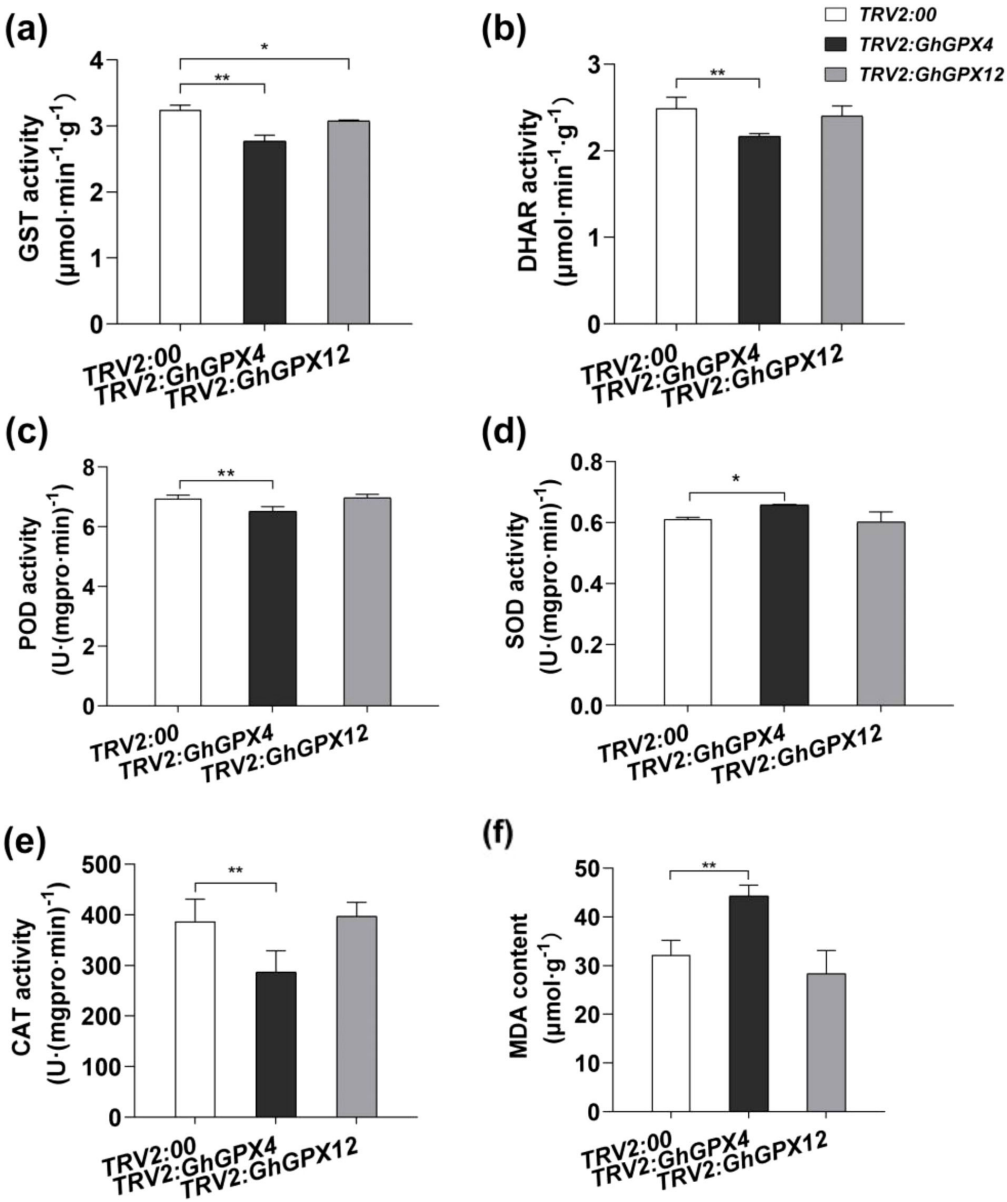
Characterisations of *GhGPX*s silencing cotton. The activity of GST **(a)**, DHAR **(b)**, POD **(c)**, SOD **(d)**, and CAT **(e)** enzyme **(f)** The detection of MDA content. *TRV2: 00* (pTRV2, negative control), *TRV2: GhGPX4* (pTRV2-*GhGPX4*), *TRV2: GhGPX12* (pTRV2-*GhGPX12*). The data are means ± SD of three biological replicates. *P< 0.05; **P<0.01 according to one-way ANOVA (Duncan’s multiple comparison test).

Results of the antioxidant enzyme activities and stress-related indicators in transgenic plants revealed that antioxidant enzyme activities (GST, DHAR, POD, CAT) in *GhGPX4* silenced cotton decreased, accompanying the levels of MDA were elevated (**Figures 13a–f**). In contrast, *GhGPX4* overexpressed *Arabidopsis* plants showed increased GST, DHAR, and POD activities (**Supplementary Figures 11a–f**). The decrease in GPX activity, along with the reduced activities of associated enzymes, such as GST and DHAR, was one of the factors contributing to the excessive accumulation of ROS during microspore development in the Jin A-CMS line.

## 4 Discussion

### 4.1 GPX family members are responsive to adverse stress conditions and ROS scavenging

Plant GPX is an antioxidant enzyme that uses thioredoxin as a reductant to catalyse the reduction of H_2_O_2_ and other organic peroxides to water or alcohols. GPX plays a key role in ROS metabolism by preventing the formation of free radicals from peroxides. Members of the *GPX* family have been identified and analysed in various plant species, including rice (5 members) (Paiva et al., 2021), wheat (14 members) (Jiang et al., 2023), *Arabidopsis* (8 members) (Rodriguez Milla et al., 2003), *Theobroma cacao* (5 members) (do Carmo Santos et al., 2024), and *G. hirsutum* (13 members) (Chen et al., 2017). Chen et al. (2017) established a basic framework for *GPX*s gene family of cotton, but their work was limited by incomplete cotton genome assemblies available then. By contrast, our study utilised high-quality annotated genomes to resolve evolutionary relationships, supplemented with in plant genetic assays, ROS quantification and protein interaction analysis, substantially refining the theoretical framework of GPX-mediated ROS regulation in cotton.

GO and KEGG enrichment revealed predominant enrichment in redox reaction and antioxidant enzyme, which coincided with our phenotypic data that GhGPX4 overexpression reduced H_2_O_2_ content and elevated antioxidant enzyme activities (**Supplementary Figures 4**, **11**). Most duplicated paralog pairs exhibited Ka/Ks < 1 (**Supplementary Table 7**), indicating dominant purifying selection constrained drastic functional alteration during evolution; minor sequence variation among duplicated genes might trigger functional divergence in ROS regulation. GPXs protect cells from oxidative damage by maintaining H_2_O_2_ homeostasis and are primarily involved in the response and regulation of abiotic stress, such as metal, cold, drought, NaCl, and oxidative stress (Miao et al., 2006; Passaia et al., 2013). The expression levels of *NnGPX* in *Nelumbo nucifera* were significantly upregulated under conditions of low temperature, heat stress, mechanical damage, and salt treatment, and its overexpression markedly enhanced salt tolerance in rice (Diao et al., 2014). In the present study, the analysis of gene expression patterns revealed that members of *GPX* family were responsive to adverse stress conditions (**Figure 7**). Specifically, *GhGPX4* was involved in ROS scavenging (**Figure 10**, **Supplementary Figure 4**).

### 4.2 New evidence suggests interactions between GhGPX4 and GhGSTs

The ascorbate-glutathione and thioredoxin systems collectively constitute the primary antioxidant defence system coupling NADPH and peroxide metabolism in plants. In the glutathione pathway, GST catalyses nucleophilic addition of the thiol group of glutathione to electrophilic molecules or substrates, such as herbicides, through nucleophilic coupling reactions. This process results in glutathionylation of the substrates, which are subsequently transported to the vacuole for degradation by ABC transporters, contributing to cellular detoxification (Philip, 1999; Cummins et al., 2013; Georgakis et al., 2021; Noctor et al., 2012).

In higher plants, the GST family can be classified into eight major categories: Phi (GSTF), Tau (GSTU), Zeta (GSTZ), Lambda (GSTL), Theta (GSTT), DHAR, tetrachlorohydroquinone dehalogenase, and elongation factor 1 gamma (Dixon et al., 2000; Seckin Dinler et al., 2023). GST is extensively involved in primary and secondary metabolism, signal transduction, and other biological processes in response to biotic and abiotic stress. GST participates in tyrosine metabolism by isomerising maleylacetoacetate and contributes to glucosinolate biosynthesis, thereby influencing plant growth and development (Dixon et al., 2000; Edwards et al., 2000; Zhang et al., 2022). Additionally, GST regulates the activity of specific regulatory proteins and enzymes by binding to various natural bioactive products or undergoing post-translational modifications. These interactions enhance the glutathione-binding activity, thereby playing a crucial role in the binding and transport of defence-related compounds in plants (Dixon et al., 2011). GST is widely present in mitochondria, chloroplasts, cell nuclei, and cytoplasm. It also functions as a GPX to counteract oxidative stress (Chen et al., 2004), a flavonoid-binding protein (Takenaka et al., 2014), a stress signalling protein (Loyall et al., 2000), a cell apoptosis factor (Kampranis et al., 2000) and plays a crucial role in endogenous metabolism. In poplars, *GSTF1* responds to salt stress by modulating xylem cell differentiation, ion homeostasis, and ROS scavenging (Gao et al., 2022). *GST* overexpression in *Arabidopsis* impairs the adverse effects that environmental stress exerts on plant metabolic activity, thereby protecting plants from oxidative damage (Qi et al., 2006).

DHAR, a specific member of the GST superfamily, is predominantly localised in plastids and features a cysteine residue instead of the active-site serine. It catalyses the glutathione-dependent reduction of dehydroascorbate to ascorbate and plays a crucial role in maintaining plant redox homeostasis (Noctor et al., 1998). DHAR provides enzymatic ligation between the ascorbate and glutathione pools (Noctor et al., 2012). The activity of antioxidant enzymes decreased (Zhang et al., 2025), resulting in oxidative stress in in Jin A-CMS.

Unlike animal GPX, which uses GSH as a reductant, plant GPX participates in redox reactions using TRX as the substrate (Noctor et al., 2012; Bela et al., 2022). Nevertheless, some studies have suggested that the glutathione and thioredoxin systems may be partially interconnected during ROS metabolism *in vivo* (Michelet et al., 2005; Reichheld et al., 2007; Marty et al., 2009). In the previous study, transcriptomic analysis revealed a significant downregulation of *GhGST* expression levels (Yang et al., 2014) and ROS levels were elevated in Jin A-CMS line (Zhang et al., 2023). Our findings using His pull-down, yeast two-hybrid, and BiFC experiments demonstrated that the GPX, GhGPX4 interacted with GSTs, including GhGSTF6 and GhDHAR2 (**Figure 11**). In contrast to chloroplast localisation of GhGPX4, the protein–protein interactions between GhGPX4 and GhGSTF6 with GhDHAR2 occurred predominantly in the nucleus and cytoplasm. This subcellular redistribution likely reflects effector-mediated modulation of protein interaction dynamics, consistent with recent reports on stress-induced relocalisation of redox regulators (Isaksson et al., 2025; Gu et al., 2022).

**Figure 14:**
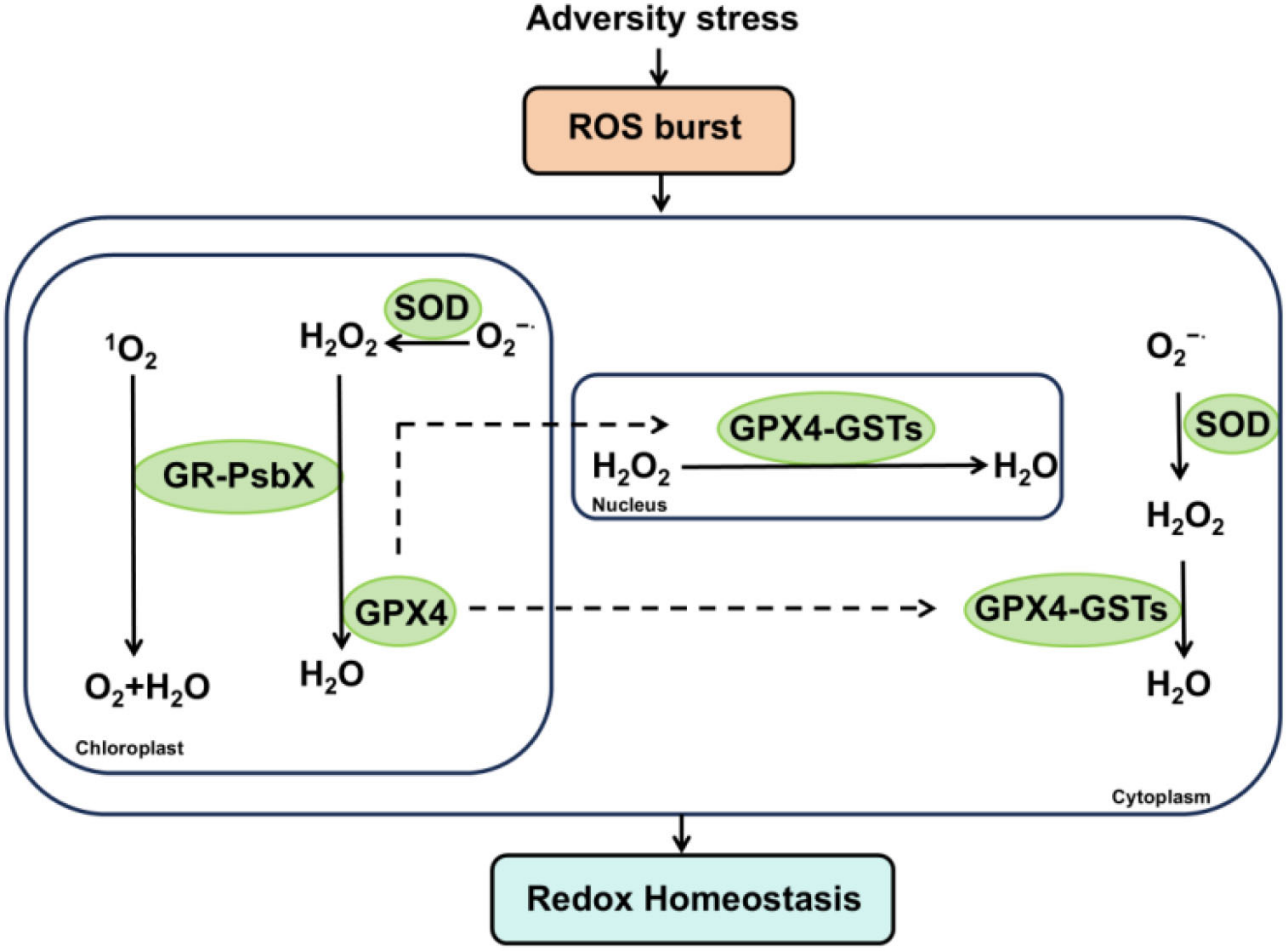
A possible working model of ROS-scavenging in Jin A-CMS. Chloroplast GPX4 and GR-PsbX complex collectively mediate ROS scavenging in chloroplasts. GPX4 also functions in cytoplasm and nucleus via interactions with GhGSTs. PsbX, photosystem II reaction center X protein.

Based on this study, we constructed a possible working model of ROS-scavenging in Jin A-CMS (**Figure 14**). In our previous finding, we found that GR participated in chloroplast ROS-scavenging via interaction with photosystem II reaction center X protein (Zhang et al., 2025). GhGPX4 mediates ROS scavenging within chloroplasts. Under the action of effector factors, GPX4 migrates to the cytoplasm and nucleus, and fulfils its physiological roles via interactions with GSTs. A greater number of associated effector factors still need to be identified in future experiments. The ascorbate-glutathione pools and thioredoxin system may be interconnected through interactions between GPX and GSTs, thereby positively regulating ROS detoxification and participating in plant redox reactions. However, the molecular mechanisms underlying these interactions require further characterisation in future research.

## 5 Conclusion

This study performed a genome-wide identification of the *GPX* gene family and characterised the functional role of GPX4 in ROS scavenging. GPX4 was predominantly localised in the chloroplast, but also involved in ROS metabolism of nucleus and cytoplasm through interactions with GSTs. The molecular mechanisms underlying these interactions should be investigated in future research.

## Data Availability

The datasets presented in this study can be found in online repositories. The names of the repository/repositories and accession number(s) can be found in the article/**Supplementary Material**.
