# Supplementary material for: Comprehensive identification of glutathione peroxidase (GPX) gene family and effect of GhGPX4 on reactive oxygen species metabolism in cotton
Source: Front Plant Sci. 2026 Jun 26;17:1846132. doi: 10.3389/fpls.2026.1846132 (PMC13350174; doi:10.3389/fpls.2026.1846132)
Supplement: Supplementary file 1 [file DataSheet1.doc]

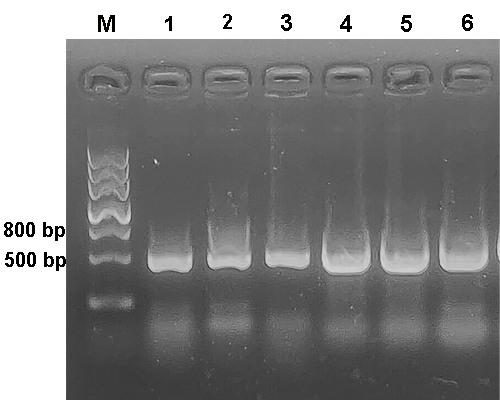
**Fig. S1.** Cloning of *GhGPXs* CDS. M, marker; 1~3, the CDS of *GhGPX4* in Jin B, Jin A, and F1, respectively; 4~6, the CDS of *GhGPX12* in Jin B, Jin A, and F1.

**
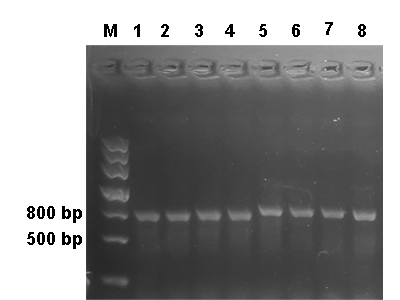
**

**Fig. S2.** PCR analysis of *GhGPXs* overexpressing *Arabidopsis*. M, marker; 1~4, *GhGPX4* overexpressing *Arabidopsis*; 5~8, *GhGPX12* overexpressing *Arabidopsis*.


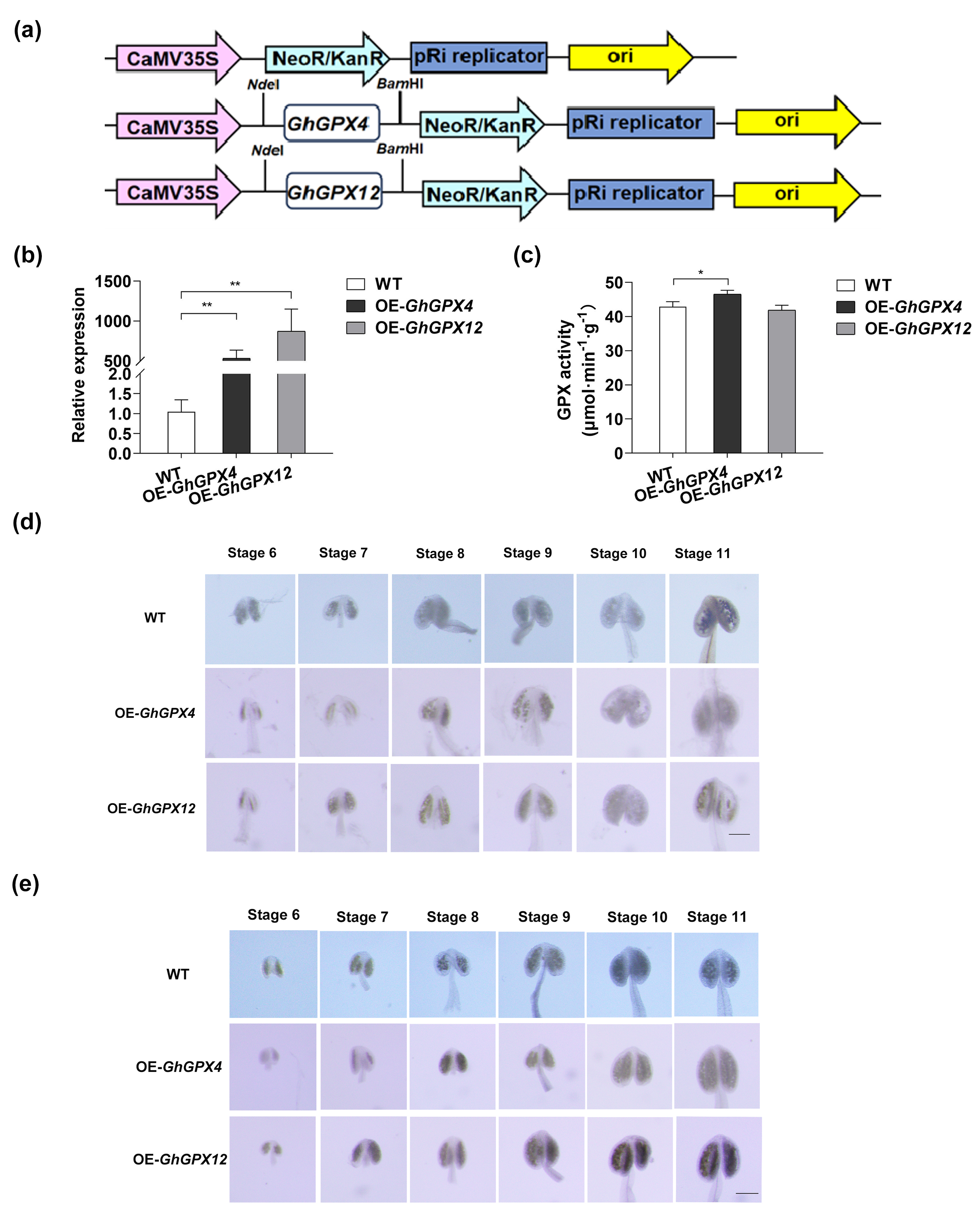


**Fig. S3.** ROS analysis of *GhGPXs* overexpressing *Arabidopsis* in anthers. The data are means ± SD of three independent experiments. *, *P<0.05*; **, *P<0.01* according to one-way ANOVA (Duncan’s multiple comparison test). (a) The recombinant vectors construction of *GhGPX*s overexpressing *Arabidopsis*. (b) The relative expression of *GhGPXs*. (c) The activity of GPX. H_2_O_2_ (d) and O₂⁻• (e) staining of *GhGPXs* overexpressing *Arabidopsis* in anthers, bar =200 μm. WT (Wild type), OE-*GhGPX4* (pRI101-*GhGPX4*), OE-*GhGPX12* (pRI101-*GhGPX12*), the data are means ± SD of three biological replicates. *, *P<0.05*; **, *P<0.01* according to one-way ANOVA (Duncan’s multiple comparison test).


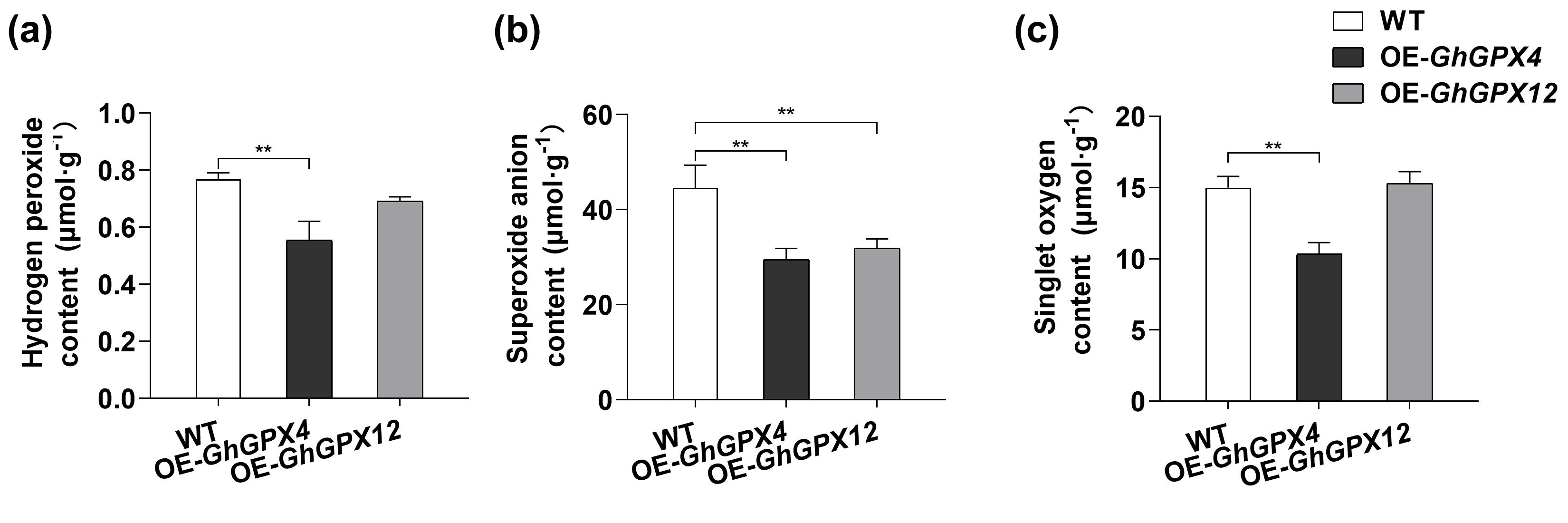


**Fig. S4.** ROS analysis of *GhGPXs* overexpressing *Arabidopsis* in leaves. The H_2_O_2_ (a), O₂^⁻•^ (b), and ^1^O_2_ (c) detection of *GhGPXs* overexpressing *Arabidopsis* in leaves. WT (Wild type), OE-*GhGPX4* (pRI101-*GhGPX4*), OE-*GhGPX12* (pRI101-*GhGPX12*), the data are means ± SD of three biological replicates. *, *P<0.05*; **, *P<0.01* according to one-way ANOVA (Duncan’s multiple comparison test).

**
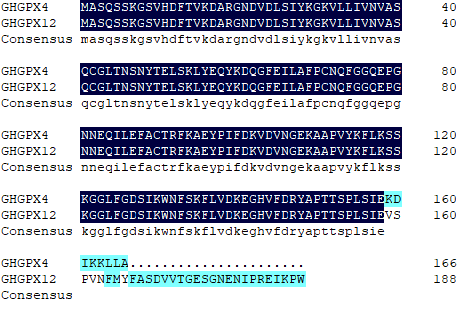
**

**Fig. S5.** Alignment of GhGPX4 with GhGPX12 protein sequences.

**
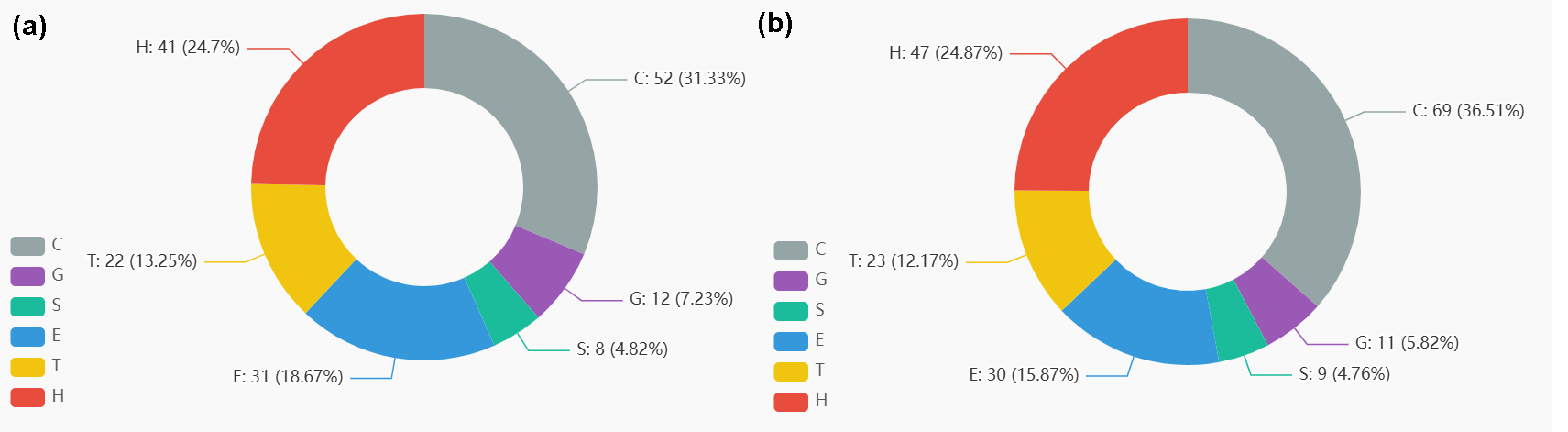
**

**Fig. S6.** Secondary structure comparison of GhGPX4 (a) with GhGPX12 (b) protein sequences.

**
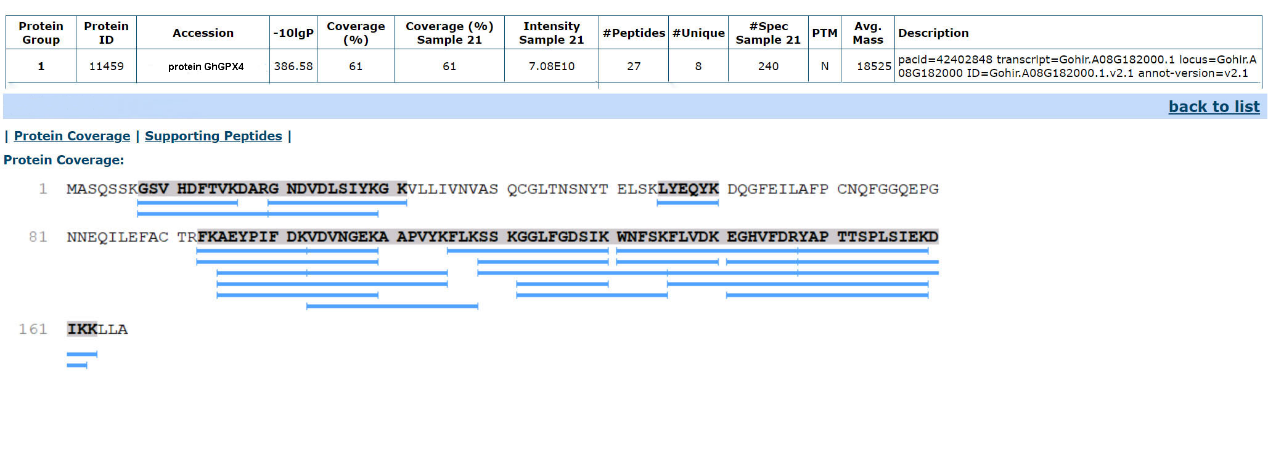
**

**Fig. S7.** Mass spectrometry identification of GhGPX4.


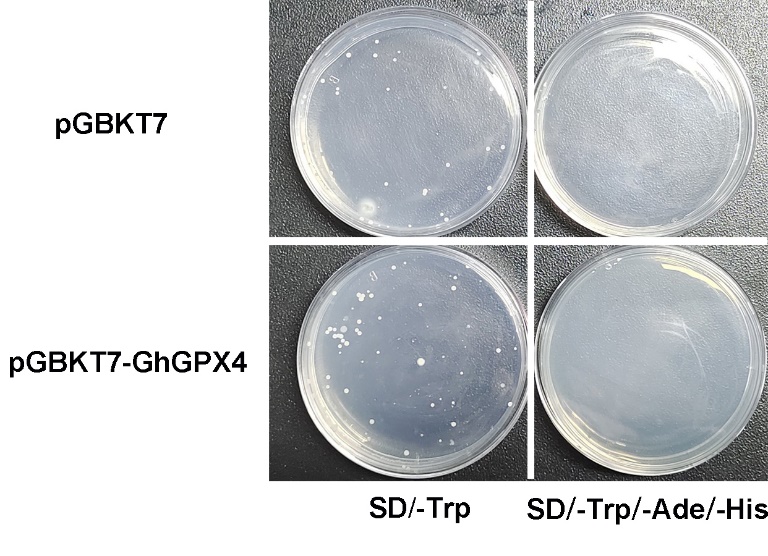


**Fig. S8.** Self-activation detection of GhGPX4.


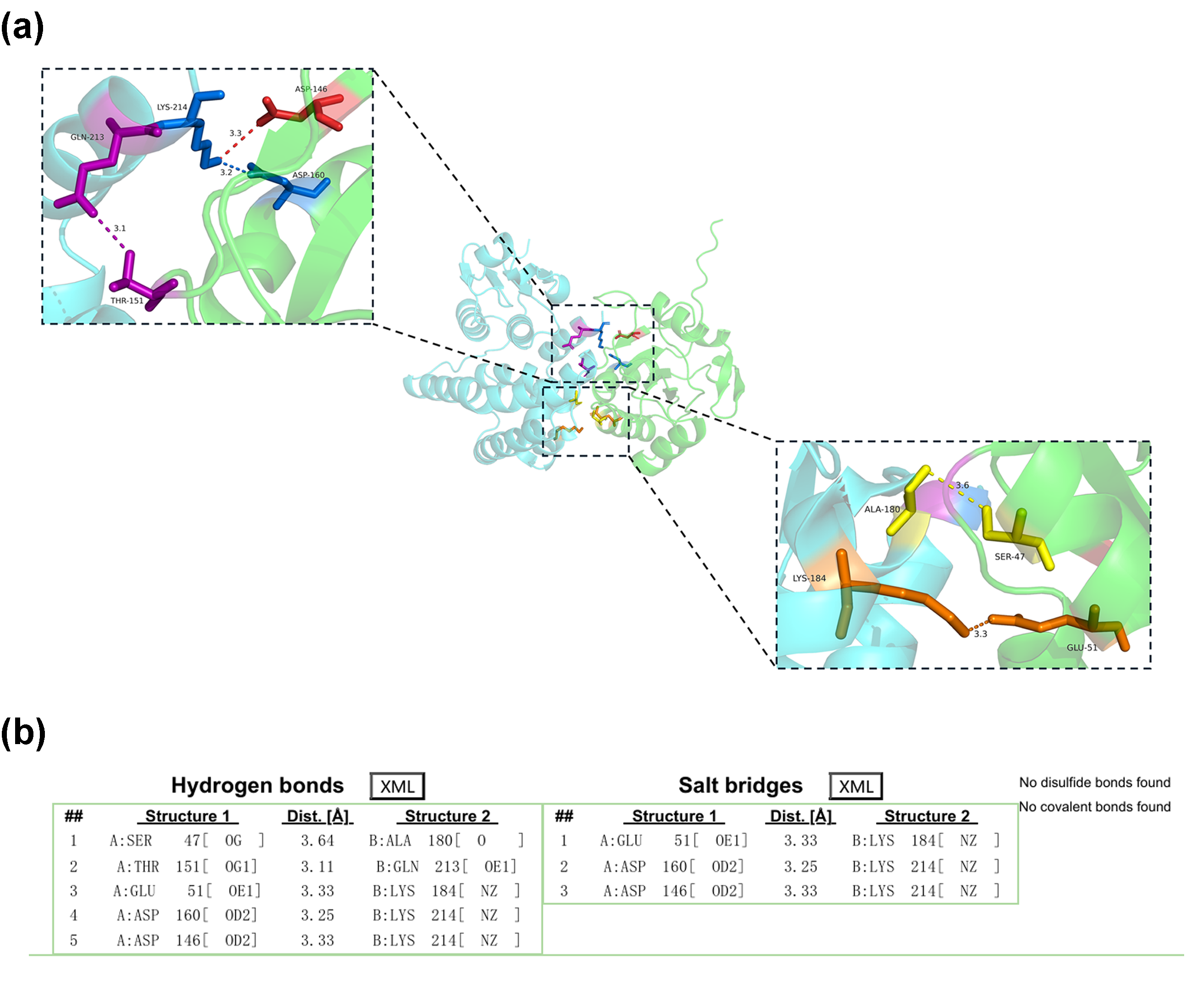


**Fig. S9.** Protein interaction binding sites. Interacting protein binding sites analysis. (a) Protein-protein docking three-dimensional structures of GhGPX4 and GhGSTF6. Green, GhGPX4; Light blue, GhGSTF6. (b) Interacting protein binding sites of GhGPX4 and GhGSTF6. Structure 1, GhGPX4; Dist., Distance; Structure 2, GhGSTF6. Structure 1, GhGPX4; Dist., Distance; Structure 2, GhDHAR2.

**
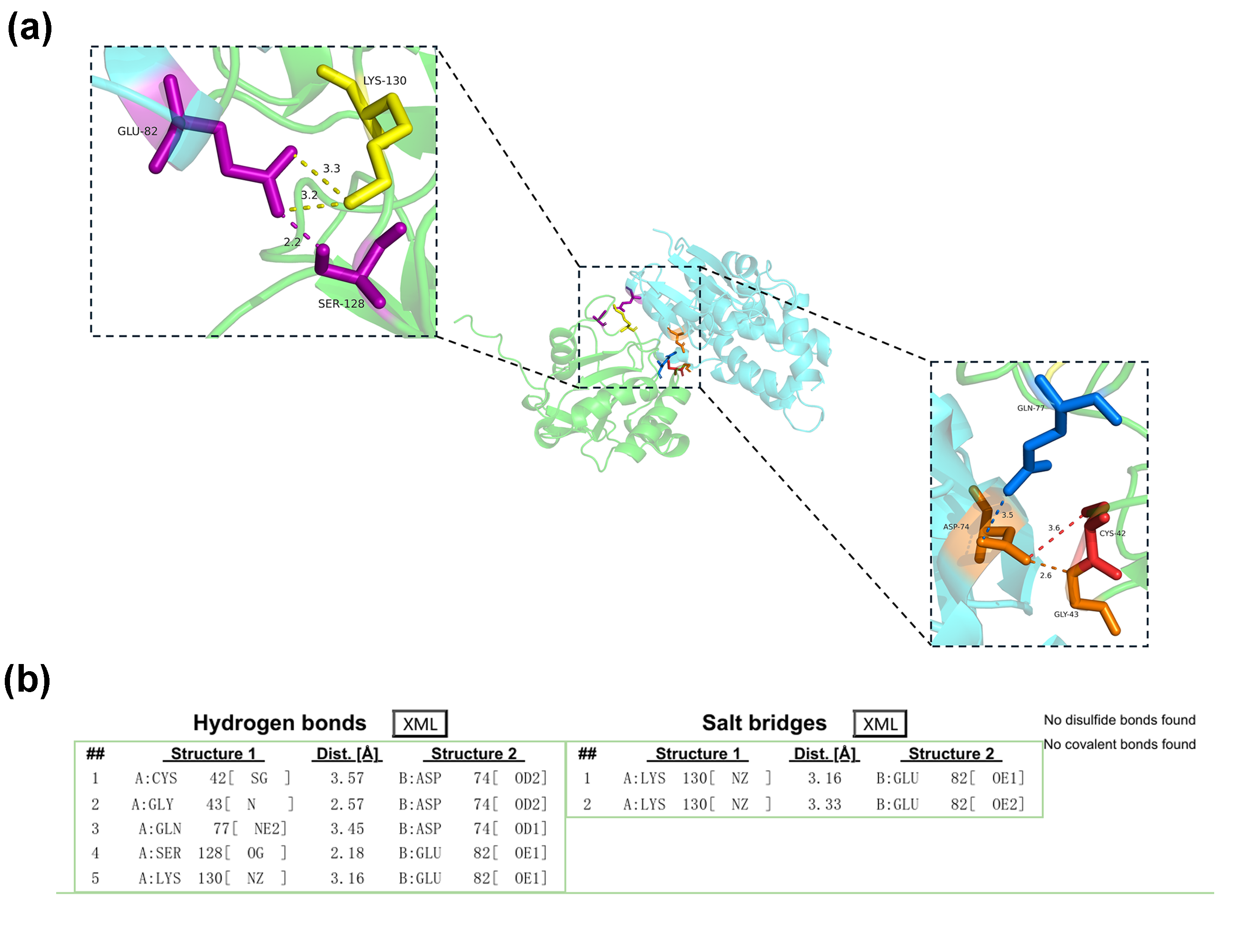
**

**Fig. S10.** Protein interaction binding sites. Interacting protein binding sites analysis. (a) Protein-protein docking three-dimensional structures of GhGPX4 and GhDHAR2. Green, GhGPX4. Light blue, GhDHAR2. (b) Interacting protein binding sites of GhGPX4 and GhDHAR2. Structure 1, GhGPX4. Dist., Distance. Structure 2, GhDHAR2.

**
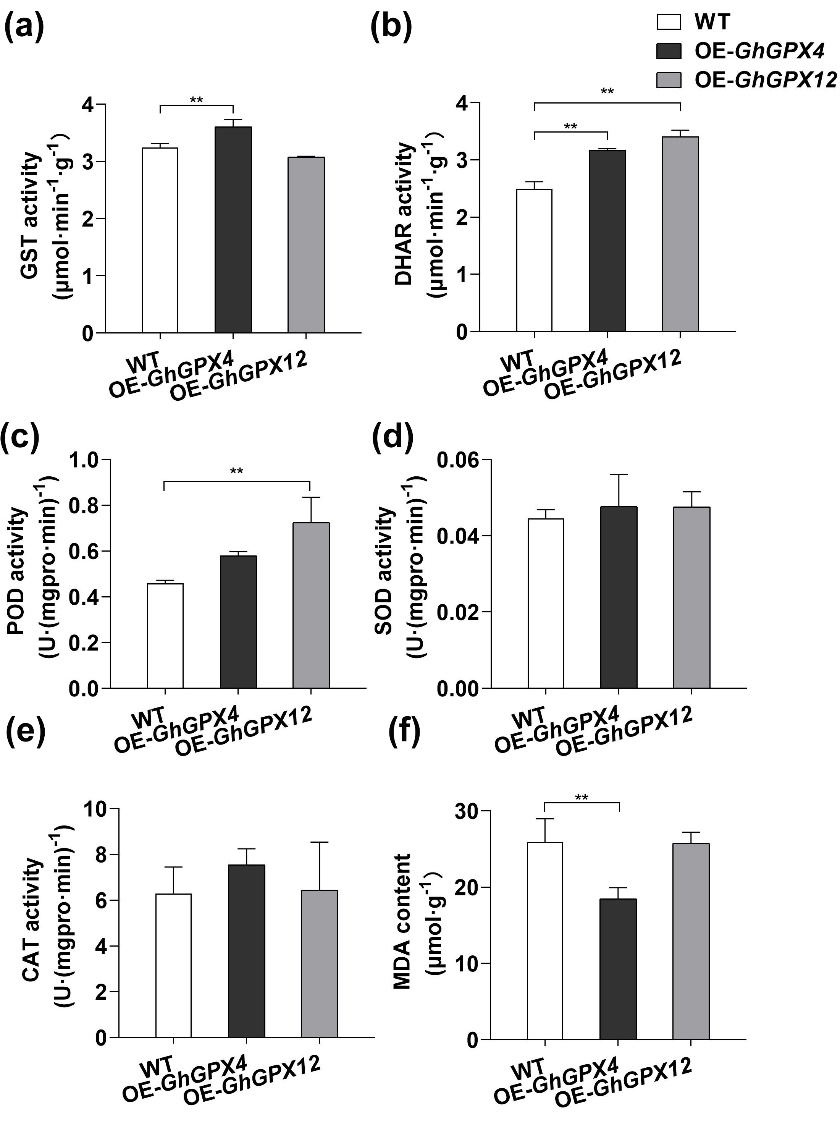
**

**Fig. S11.** Determination of antioxidant enzyme activity in *GhGPXs* overexpressing *Arabidopsis*. The activity of GST (a), DHAR (b), POD (c), SOD (d), CAT (e). (f) The detection of MDA content. *TRV2: 00* (pTRV2, negative control), *TRV2: GhGPX4* (pTRV2-*GhGPX4*), *TRV2: GhGPX12* (pTRV2-*GhGPX12*)*.* The data are means ± SD of three biological replicates. *, *P<0.05*; **, *P<0.01* according to one-way ANOVA (Duncan’s multiple comparison test).

**Table S1.** Primer list.

| **Primer Name** | **Sequence** |
| --- | --- |
| **GhGPX4-qPCR-F** | TAGAGTTTGCTTGCACTCGC |
| **GhGPX4-qPCR-R** | CTGTCCCCAAAAAGTCCACC |
| **GhGPX12-qPCR-F** | GGGCCATGTCTTCGATCGTT |
| **GhGPX12-qPCR-R** | CCCTTGGGATGTTCTCATTTCC |
| **GhGPX4-F** | TCGCCTGATTCCGCTGTTAT |
| **GhGPX4-R** | ACTCATCAAATTCAAGCCAGCA |
| **GhGPX12-F** | ATGGCTTCTCAATCTTCTA |
| **GhGPX12-R** | TCAATTCCAGGGTTAATTTC |
| **GhGPX4-pCAMBIA1302-F** | GGACTCTTGACCATGGATGGCTTCTCAACCTTCTAAGGGA |
| **GhGPX4-pCAMBIA1302-R** | GTACCGAGCTCCATGGCCAGCAGTTTCTTTATATCCTTCTCAATG |
| **GhGPX12-pCAMBIA1302-F** | GGACTCTTGACCATGGATGGCTTCTCAACCTTCTAAGGGA |
| **GhGPX12-pCAMBIA1302-R** | GTACCGAGCTCCATGTTCCAGGGTTTAATTTCCCTTGGGATGTT |
| **GhGPX4-pRI101-F** | TTCAGAATTCGGATCTCAATTCCAGGGTTTAATTTCCCTTGGG |
| **GhGPX4-pRI101-R** | CACTGTTGATACATAATGGCTTCTCAATCTTCTAAGGGATCAG |
| **GhGPX12-pRI101-F** | TTCAGAATTCGGATCTCAATTCCAGGGTTTAATTTC |
| **GhGPX12-pRI101-R** | CACTGTTGATACATAATGGCATGGCTTCTCAATCTTCTA |
| **GhGPX4-pTRV2-F** | TAAGGTTACCGAATTCCAATGTGGCTTGACCAATTCCAACT |
| **GhGPX4-pTRV2-R** | GCTCGGTACCGGATCCTCAAGCCAGCAGTTTCTTTATATCCTTCTCA |
| **GhGPX12-pTRV2-F** | TAAGGTTACCGAATTCTATAAAGATCAAGGTTTTGAGATTCTTGCATTCCC |
| **GhGPX12-pTRV2-R** | GCTCGGTACCGGATCCTCAATTCCAGGGTTTAATTTCCCTTGGG |
| **GhGPX4-pET22b-F** | GTGCGGCCGCAAGCTCCAGCAGTTTCTTTATATCCTTCTCAATG |
| **GhGPX4-pET22b-R** | GAATTAATTCGGATCGAATGGCTTCTCAACCTTCTAAG |
| **GhGPX4-PSY-F** | CTGGCGCGCCACTAGTGGATCATGGCTTCTCAACCTTCTAAGG |
| **GhGPX4-PSY-R** | TCCCGGGAGCGGTACCCTCGAAAGCCAGCAGTTTCTTTATATCC |
| **GhGPX4-pGBKT7-F** | CATGGAGGCCGAATTAATGGCTTCTCAACCTTCTAAGG |
| **GhGPX4-pGBKT7-R** | GCAGGTCGACGGATCTCAAGCCAGCAGTTTCTTTATATCC |
| **GhGSTF6-F** | CTCATCGAACAACTATGGCAGC |
| **GhGSTF6-R** | TCCAAATCGCCGAAGCCTTT |
| **GhDHAR2-F** | ATCAAACCCAGAAAGCTCAAAGG |
| **GhDHAR2-R** | ACACCTCACTGTTTCCAGTTTG |
| **GhGSTF6-PSY-F** | CTGGCGCGCCACTAGTGGATCATGGCAGCCATCAAAGTC |
| **GhGSTF6-PSY-R** | TCCCGGGAGCGGTACCCTCGAACTGTTTTTGCATAGCGAGG |
| **GhDHAR2-PSY-F** | CTGGCGCGCCACTAGTGGATCATGGCTTTGGAGATCTGTGT |
| **GhDHAR2-PSY-R** | TCCCGGGAGCGGTACCCTCGAATGCATTCACCTTAGGTGC |
| **GhDHAR2-pGADT7-F** | GGAGGCCAGTGAATTAATGGCTTTGGAGATCTGTG |
| **GhDHAR2-pGADT7-R** | TCATCTGCAGCTCGATCATGCATTCACCTTAGGTG |
| **GhGSTF6-pGADT7-F** | GGAGGCCAGTGAATTAATGGCAGCCATCAAAGT |
| **GhGSTF6-pGADT7-R** | TCATCTGCAGCTCGATCACTGTTTTTGCATAGCGAG |
| **GhEF1α-F** | AGACCACCAAGTACTACTGCAC |
| **GhEF1α-R** | CCACCAATCTTGTACACATCC |
| **AtActin8-F** | ATGACTCAGATCATGTTTGAGACC |
| **AtActin8-R** | TCAGTAAGGTCACGACCAGCAA |

**Table S2.** GPXs sequence information.

| **locus names** | **gene names** | **ORF**  **(bp)** | **AA length** | **MW**  **(kDa)** | **pI** | **Instability index** | **Aliphatic index** | **GRAVY** | **signal peptide** |
| --- | --- | --- | --- | --- | --- | --- | --- | --- | --- |
| **Ghir_A07G003740** | **GhGPX1** | 729 | 242 | 26.74 | 9.73 | 33.62 | 70.12 | -0.228 | No |
| **Ghir_A08G007460** | **GhGPX2** | 516 | 171 | 19.17 | 9.50 | 30.90 | 71.23 | -0.469 | No |
| **Ghir_A08G007910** | **GhGPX3** | 627 | 208 | 23.73 | 5.36 | 39.21 | 77.31 | -0.310 | Yes |
| **Ghir_A08G019020** | **GhGPX4** | 501 | 166 | 18.53 | 7.34 | 28.13 | 76.93 | -0.382 | No |
| **Ghir_A08G019410** | **GhGPX5** | 516 | 171 | 19.22 | 8.43 | 28.90 | 74.68 | -0.463 | No |
| **Ghir_A09G017710** | **GhGPX6** | 528 | 175 | 19.90 | 8.44 | 23.21 | 82.46 | -0.298 | No |
| **Ghir_A12G024110** | **GhGPX7** | 666 | 221 | 24.52 | 9.63 | 35.45 | 85.57 | -0.250 | Yes |
| **Ghir_A12G024130** | **GhGPX8** | 507 | 168 | 19.29 | 4.84 | 29.70 | 81.19 | -0.399 | No |
| **Ghir_D07G003760** | **GhGPX9** | 729 | 242 | 26.66 | 9.73 | 36.16 | 68.51 | -0.241 | No |
| **Ghir_D08G007550** | **GhGPX10** | 384 | 127 | 14.33 | 9.38 | 33.35 | 65.98 | -0.556 | No |
| **Ghir_D08G007950** | **GhGPX11** | 627 | 208 | 23.67 | 5.85 | 37.38 | 79.66 | -0.251 | Yes |
| **Ghir_D08G019910** | **GhGPX12** | 570 | 189 | 21.50 | 9.46 | 30.62 | 83.02 | -0.271 | No |
| **Ghir_D08G020300** | **GhGPX13** | 516 | 171 | 19.19 | 9.01 | 27.66 | 74.09 | -0.462 | No |
| **Ghir_D09G017200** | **GhGPX14** | 426 | 141 | 15.83 | 5.28 | 26.53 | 81.56 | -0.318 | No |
| **Ghir_D12G024110** | **GhGPX15** | 702 | 233 | 25.49 | 8.75 | 35.32 | 90.39 | -0.171 | Yes |
| **Ghir_D12G024130** | **GhGPX16** | 507 | 168 | 19.13 | 4.33 | 30.12 | 80.00 | -0.341 | No |
| **Grai_07G025450** | **GrGPX1** | 729 | 242 | 26.69 | 9.30 | 36.16 | 68.51 | -0.243 | No |
| **Grai_08G008400** | **GrGPX2** | 528 | 175 | 19.97 | 9.24 | 32.99 | 70.69 | -0.402 | No |
| **Grai_08G008910** | **GrGPX3** | 627 | 208 | 23.66 | 5.51 | 37.97 | 77.79 | -0.276 | Yes |
| **Grai_08G024280** | **GrGPX4** | 501 | 166 | 18.49 | 6.73 | 25.78 | 79.28 | -0.372 | No |
| **Grai_08G024730** | **GrGPX5** | 516 | 171 | 19.29 | 8.64 | 28.29 | 74.09 | -0.484 | No |
| **Grai_09G020450** | **GrGPX6** | 507 | 168 | 18.79 | 6.73 | 29.86 | 80.65 | -0.326 | No |
| **Grai_12G027780** | **GrGPX7** | 753 | 250 | 27.46 | 6.90 | 38.88 | 87.40 | -0.127 | Yes |
| **Grai_12G027800** | **GrGPX8** | 507 | 168 | 19.13 | 4.59 | 30.12 | 80.00 | -0.341 | No |
| **Garb_07G025980** | **GaGPX1** | 729 | 242 | 26.77 | 9.29 | 33.62 | 70.12 | -0.230 | No |
| **Garb_08G008190** | **GaGPX2** | 516 | 171 | 19.22 | 8.32 | 28.90 | 74.68 | -0.463 | No |
| **Garb_08G008600** | **GaGPX3** | 501 | 166 | 18.53 | 6.73 | 28.13 | 76.93 | -0.382 | No |
| **Garb_08G024070** | **GaGPX4** | 660 | 219 | 25.20 | 5.97 | 35.01 | 76.99 | -0.325 | Yes |
| **Garb_08G024650** | **GaGPX5** | 507 | 168 | 18.75 | 9.22 | 33.73 | 73.63 | -0.490 | No |
| **Garb_09G021030** | **GaGPX6** | 570 | 189 | 21.45 | 9.11 | 30.17 | 81.48 | -0.296 | No |
| **Garb_12G028330** | **GaGPX7** | 702 | 233 | 25.50 | 8.82 | 36.38 | 90.39 | -0.171 | Yes |
| **Garb_12G028350** | **GaGPX8** | 507 | 168 | 19.19 | 4.84 | 31.61 | 81.19 | -0.372 | No |

**Table S3.** GhGPXs sequence alignment information with Chen et al. (2017).

| **This study (HAU_v1.1)** |  | **Chen et al. (2017, HAU_v2.0)** |  |  |
| --- | --- | --- | --- | --- |
| **Gene ID** | **Chr** | **Gene ID** | **Chr** | **Note** |
| **Ghir_A07G003740.1** | A07 | CotAD_10469 | Dt_chr1 | Conserved |
| **Ghir_A08G007460.1** | A08 | CotAD_76066, CotAD_54994, CotAD_36707 | Dt_chr4, At_chr4, scaffold | Conserved |
| **Ghir_A08G007910.1** | A08 |  |  | New in v1.1 |
| **Ghir_A08G019020.1** | A08 | CotAD_22672, CotAD_58608, CotAD_39880, CotAD_59095, CotAD_51884 | Dt_chr4, At_chr4, Dt_chr8, At_chr10, scaffold | Conserved |
| **Ghir_A08G019410.1** | A08 | CotAD_76066, CotAD_54994, CotAD_36707 | Dt_chr4, At_chr4, scaffold | Conserved |
| **Ghir_A09G017710.1** | A09 |  |  | New in v1.1 |
| **Ghir_A12G024110.1** | A12 | Gh_A12G2084 | A12 | Conserved |
| **Ghir_A12G024130.1** | A12 | CotAD_39878, CotAD_39520 | Dt_chr8, At_chr13 | Conserved |
| **Ghir_D07G003760.1** | D07 | CotAD_10469 | Dt_chr1 | Conserved |
| **Ghir_D08G007550.1** | D08 | CotAD_76066, CotAD_54994, CotAD_36707 | Dt_chr4, At_chr4, scaffold | Conserved |
| **Ghir_D08G007950.1** | D08 |  |  | New in v1.1 |
| **Ghir_D08G019910.1** | D08 |  |  | New in v1.1 |
| **Ghir_D08G020300.1** | D08 | CotAD_76066, CotAD_54994, CotAD_36707 | Dt_chr4, At_chr4, scaffold | Conserved |
| **Ghir_D09G017200.1** | D09 |  |  | New in v1.1 |
| **Ghir_D12G024110.1** | D12 | Gh_D12G2260 | D12 | Conserved |
| **Ghir_D12G024130.1** | D12 | CotAD_39878, CotAD_39520 | Dt_chr8, At_chr13 | Conserved |

**Table S4.** Cis-elements present in the promoters of *GhGPX*s.

| **Gene cis-element** | **Annotation** | **GhGPX1** | **GhGPX2** | **GhGPX3** | **GhGPX4** | **GhGPX5** | **GhGPX6** | **GhGPX7** | **GhGPX8** | **GhGPX9** | **GhGPX10** | **GhGPX11** | **GhGPX12** | **GhGPX13** | **GhGPX14** | **GhGPX15** | **GhGPX16** |
| --- | --- | --- | --- | --- | --- | --- | --- | --- | --- | --- | --- | --- | --- | --- | --- | --- | --- |
| **TCT-motif** | photoresponse | 2 | 1 | 1 | 0 | 0 | 0 | 0 | 0 | 3 | 1 | 1 | 0 | 0 | 2 | 0 | 0 |
| **GA-motif** | photoresponse | 1 | 0 | 1 | 1 | 0 | 0 | 0 | 0 | 1 | 1 | 1 | 0 | 0 | 0 | 0 | 0 |
| **G-box** | photoresponse | 1 | 1 | 1 | 1 | 3 | 1 | 5 | 3 | 1 | 0 | 1 | 0 | 1 | 4 | 5 | 4 |
| **GT1-motif** | photoresponse | 5 | 0 | 2 | 2 | 1 | 1 | 1 | 1 | 3 | 1 | 2 | 2 | 0 | 1 | 0 | 1 |
| **Box 4** | photoresponse | 3 | 2 | 9 | 2 | 4 | 3 | 0 | 1 | 2 | 0 | 9 | 3 | 2 | 3 | 0 | 1 |
| **ATC-motif** | photoresponse | 0 | 0 | 0 | 0 | 0 | 0 | 0 | 0 | 0 | 0 | 0 | 0 | 1 | 0 | 0 | 0 |
| **AE-box** | photoresponse | 1 | 0 | 0 | 0 | 0 | 0 | 1 | 1 | 1 | 1 | 1 | 0 | 0 | 0 | 0 | 1 |
| **Sp1** | photoresponse | 0 | 1 | 0 | 0 | 0 | 1 | 0 | 0 | 0 | 0 | 0 | 0 | 0 | 1 | 0 | 0 |
| **AT1-motif** | photoresponse | 0 | 0 | 1 | 0 | 0 | 0 | 0 | 0 | 0 | 0 | 0 | 0 | 1 | 0 | 0 | 1 |
| **chs-CMA1a** | photoresponse | 0 | 0 | 1 | 0 | 0 | 1 | 0 | 0 | 0 | 0 | 1 | 1 | 0 | 0 | 0 | 0 |
| **LAMP-element** | photoresponse | 0 | 0 | 1 | 0 | 0 | 0 | 0 | 0 | 0 | 0 | 0 | 0 | 0 | 0 | 1 | 0 |
| **GATA-motif** | photoresponse | 0 | 0 | 0 | 2 | 0 | 0 | 1 | 0 | 0 | 0 | 0 | 0 | 0 | 0 | 3 | 0 |
| **ATCT-motif** | photoresponse | 0 | 0 | 0 | 2 | 1 | 0 | 0 | 1 | 0 | 0 | 0 | 1 | 0 | 2 | 0 | 1 |
| **TCCC-motif** | photoresponse | 0 | 0 | 0 | 0 | 0 | 1 | 0 | 0 | 0 | 0 | 1 | 0 | 0 | 2 | 0 | 0 |
| **ACE** | photoresponse | 0 | 0 | 0 | 0 | 0 | 0 | 1 | 0 | 0 | 1 | 0 | 0 | 1 | 1 | 1 | 0 |
| **3-AF1 binding site** | photoresponse | 0 | 0 | 0 | 0 | 0 | 0 | 1 | 0 | 0 | 0 | 0 | 0 | 0 | 0 | 1 | 0 |
| **Box II** | photoresponse | 0 | 0 | 0 | 0 | 0 | 0 | 0 | 0 | 0 | 1 | 0 | 0 | 0 | 0 | 0 | 0 |
| **I-box** | photoresponse | 0 | 0 | 0 | 0 | 0 | 0 | 0 | 0 | 0 | 0 | 0 | 1 | 1 | 1 | 0 | 0 |
| **MARE** | photoresponse | 0 | 0 | 0 | 0 | 2 | 0 | 0 | 0 | 0 | 0 | 0 | 0 | 0 | 0 | 0 | 0 |
| **MRE** | photoresponse | 0 | 0 | 0 | 0 | 0 | 0 | 1 | 0 | 0 | 0 | 0 | 0 | 1 | 0 | 0 | 0 |
| **P-box** | phytohormone | 1 | 0 | 0 | 0 | 0 | 0 | 1 | 0 | 0 | 2 | 0 | 0 | 0 | 0 | 1 | 0 |
| **GARE-motif** | phytohormone | 0 | 0 | 1 | 0 | 0 | 0 | 0 | 0 | 0 | 0 | 0 | 0 | 1 | 0 | 0 | 0 |
| **ABRE** | phytohormone | 2 | 0 | 0 | 1 | 5 | 1 | 3 | 3 | 1 | 0 | 0 | 0 | 1 | 4 | 4 | 3 |
| **TATC-box** | phytohormone | 1 | 0 | 0 | 0 | 0 | 0 | 0 | 0 | 1 | 0 | 0 | 0 | 0 | 0 | 0 | 0 |
| **AuxRR-core** | phytohormone | 1 | 0 | 0 | 0 | 0 | 0 | 0 | 0 | 1 | 0 | 0 | 0 | 0 | 0 | 0 | 0 |
| **TGA-element** | phytohormone | 0 | 2 | 2 | 1 | 0 | 0 | 1 | 0 | 0 | 0 | 0 | 0 | 0 | 0 | 1 | 0 |
| **CGTCA-motif** | phytohormone | 0 | 2 | 2 | 3 | 2 | 0 | 0 | 1 | 1 | 2 | 1 | 1 | 0 | 1 | 0 | 1 |
| **TGACG-motif** | phytohormone | 0 | 2 | 2 | 3 | 2 | 0 | 0 | 1 | 1 | 2 | 1 | 1 | 0 | 1 | 0 | 1 |
| **TCA-element** | phytohormone | 0 | 0 | 1 | 0 | 0 | 0 | 1 | 1 | 0 | 0 | 1 | 0 | 1 | 1 | 0 | 0 |
| **ARE** | stress | 5 | 0 | 2 | 1 | 1 | 1 | 3 | 3 | 4 | 4 | 2 | 2 | 0 | 0 | 4 | 4 |
| **MBS** | stress | 2 | 0 | 0 | 1 | 0 | 1 | 1 | 0 | 4 | 0 | 0 | 0 | 0 | 1 | 1 | 0 |
| **LTR** | stress | 0 | 1 | 0 | 1 | 0 | 0 | 0 | 0 | 0 | 2 | 0 | 0 | 1 | 0 | 0 | 0 |
| **circadian** | stress | 0 | 0 | 1 | 0 | 0 | 0 | 0 | 0 | 0 | 0 | 0 | 1 | 0 | 0 | 0 | 1 |
| **TC-rich repeats** | stress | 0 | 0 | 0 | 0 | 0 | 0 | 0 | 1 | 1 | 0 | 1 | 1 | 0 | 1 | 0 | 0 |

**Table S5.** GO terms analysis.

|  | **GO ID** | **GO Term** | **GeneID** |
| --- | --- | --- | --- |
| **Biological Process (BP)** | GO:0006979 | response to oxidative stress | GhGPX1; GhGPX2; GhGPX3; GhGPX4; GhGPX5; GhGPX6; GhGPX7; GhGPX8; GhGPX9; GhGPX10; GhGPX11; GhGPX12; GhGPX13; GhGPX14; GhGPX15; GhGPX16 |
|  | GO:0055114 | oxidation-reduction process | GhGPX1; GhGPX2; GhGPX3; GhGPX4; GhGPX5; GhGPX6; GhGPX7; GhGPX8; GhGPX9; GhGPX10; GhGPX11; GhGPX12; GhGPX13; GhGPX14; GhGPX15; GhGPX16 |
|  | GO:0006950 | response to stress | GhGPX1; GhGPX2; GhGPX3; GhGPX4; GhGPX5; GhGPX6; GhGPX7; GhGPX8; GhGPX9; GhGPX10; GhGPX11; GhGPX12; GhGPX13; GhGPX14; GhGPX15; GhGPX16 |
|  | GO:0009407 | toxin catabolic process | GhGPX3; GhGPX6; GhGPX8; GhGPX11; GhGPX14; GhGPX16 |
|  | GO:0090487 | secondary metabolite catabolic process | GhGPX3; GhGPX6; GhGPX8; GhGPX11; GhGPX14; GhGPX16 |
|  | GO:0009404 | toxin metabolic process | GhGPX3; GhGPX6; GhGPX8; GhGPX11; GhGPX14; GhGPX16 |
|  | GO:0050896 | response to stimulus | GhGPX1; GhGPX2; GhGPX3; GhGPX4; GhGPX5; GhGPX6; GhGPX7; GhGPX8; GhGPX9; GhGPX10; GhGPX11; GhGPX12; GhGPX13; GhGPX14; GhGPX15; GhGPX16 |
|  | GO:0042631 | cellular response to water deprivation | GhGPX3; GhGPX6; GhGPX11; GhGPX14 |
|  | GO:0071462 | cellular response to water stimulus | GhGPX3; GhGPX6; GhGPX11; GhGPX14 |
|  | GO:0044710 | single-organism metabolic process | GhGPX1; GhGPX2; GhGPX3; GhGPX4; GhGPX5; GhGPX6; GhGPX7; GhGPX8; GhGPX9; GhGPX10; GhGPX11; GhGPX12; GhGPX13; GhGPX14; GhGPX15; GhGPX16 |
|  | GO:0010035 | response to inorganic substance | GhGPX3; GhGPX4; GhGPX6; GhGPX7; GhGPX11; GhGPX12; GhGPX14; GhGPX15 |
|  | GO:0019748 | secondary metabolic process | GhGPX3; GhGPX6; GhGPX8; GhGPX11; GhGPX14; GhGPX16 |
|  | GO:0071214 | cellular response to abiotic stimulus | GhGPX3; GhGPX6; GhGPX11; GhGPX14 |
|  | GO:0042542 | response to hydrogen peroxide | GhGPX3; GhGPX6; GhGPX11; GhGPX14 |
|  | GO:0009738 | abscisic acid-activated signaling pathway | GhGPX3; GhGPX6; GhGPX11; GhGPX14 |
|  | GO:0071215 | cellular response to abscisic acid stimulus | GhGPX3; GhGPX6; GhGPX11; GhGPX14 |
|  | GO:0031668 | cellular response to extracellular stimulus | GhGPX3; GhGPX6; GhGPX11; GhGPX14 |
|  | GO:0071496 | cellular response to external stimulus | GhGPX3; GhGPX6; GhGPX11; GhGPX14 |
|  | GO:0009991 | response to extracellular stimulus | GhGPX3; GhGPX6; GhGPX11; GhGPX14 |
|  | GO:0097306 | cellular response to alcohol | GhGPX3; GhGPX6; GhGPX11; GhGPX14 |
|  | GO:0000302 | response to reactive oxygen species | GhGPX3; GhGPX6; GhGPX11; GhGPX14 |
|  | GO:0071396 | cellular response to lipid | GhGPX3; GhGPX6; GhGPX11; GhGPX14 |
|  | GO:0009414 | response to water deprivation | GhGPX3; GhGPX6; GhGPX11; GhGPX14 |
|  | GO:0044712 | single-organism catabolic process | GhGPX3; GhGPX6; GhGPX8; GhGPX11; GhGPX14; GhGPX16 |
|  | GO:0009628 | response to abiotic stimulus | GhGPX3; GhGPX4; GhGPX6; GhGPX7; GhGPX11; GhGPX12; GhGPX14; GhGPX15 |
|  | GO:0009415 | response to water | GhGPX3; GhGPX6; GhGPX11; GhGPX14 |
|  | GO:0071229 | cellular response to acid chemical | GhGPX3; GhGPX6; GhGPX11; GhGPX14 |
|  | GO:0044248 | cellular catabolic process | GhGPX3; GhGPX6; GhGPX8; GhGPX11; GhGPX14; GhGPX16 |
|  | GO:0009793 | embryo development ending in seed dormancy | GhGPX2; GhGPX5; GhGPX10; GhGPX13 |
|  | GO:0009737 | response to abscisic acid | GhGPX3; GhGPX6; GhGPX11; GhGPX14 |
|  | GO:0044699 | single-organism process | GhGPX1; GhGPX2; GhGPX3; GhGPX4; GhGPX5; GhGPX6; GhGPX7; GhGPX8; GhGPX9; GhGPX10; GhGPX11; GhGPX12; GhGPX13; GhGPX14; GhGPX15; GhGPX16 |
|  | GO:0046686 | response to cadmium ion | GhGPX4; GhGPX7; GhGPX12; GhGPX15 |
|  | GO:0042221 | response to chemical | GhGPX3; GhGPX4; GhGPX6; GhGPX7; GhGPX11; GhGPX12; GhGPX14; GhGPX15 |
|  | GO:1901701 | cellular response to oxygen-containing compound | GhGPX3; GhGPX6; GhGPX11; GhGPX14 |
|  | GO:0097305 | response to alcohol | GhGPX3; GhGPX6; GhGPX11; GhGPX14 |
|  | GO:0048316 | seed development | GhGPX2; GhGPX5; GhGPX10; GhGPX13 |
|  | GO:0009790 | embryo development | GhGPX2; GhGPX5; GhGPX10; GhGPX13 |
|  | GO:0009755 | hormone-mediated signaling pathway | GhGPX3; GhGPX6; GhGPX11; GhGPX14 |
|  | GO:0032870 | cellular response to hormone stimulus | GhGPX3; GhGPX6; GhGPX11; GhGPX14 |
|  | GO:0071495 | cellular response to endogenous stimulus | GhGPX3; GhGPX6; GhGPX11; GhGPX14 |
|  | GO:0010038 | response to metal ion | GhGPX4; GhGPX7; GhGPX12; GhGPX15 |
|  | GO:0010154 | fruit development | GhGPX2; GhGPX5; GhGPX10; GhGPX13 |
|  | GO:0033993 | response to lipid | GhGPX3; GhGPX6; GhGPX11; GhGPX14 |
|  | GO:0009651 | response to salt stress | GhGPX4; GhGPX7; GhGPX12; GhGPX15 |
|  | GO:0009056 | catabolic process | GhGPX3; GhGPX6; GhGPX8; GhGPX11; GhGPX14; GhGPX16 |
|  | GO:0006970 | response to osmotic stress | GhGPX4; GhGPX7; GhGPX12; GhGPX15 |
|  | GO:0071310 | cellular response to organic substance | GhGPX3; GhGPX6; GhGPX11; GhGPX14 |
|  | GO:0070887 | cellular response to chemical stimulus | GhGPX3; GhGPX6; GhGPX11; GhGPX14 |
|  | GO:0033554 | cellular response to stress | GhGPX3; GhGPX6; GhGPX11; GhGPX14 |
|  | GO:0001101 | response to acid chemical | GhGPX3; GhGPX6; GhGPX11; GhGPX14 |
|  | GO:0008152 | metabolic process | GhGPX1; GhGPX2; GhGPX3; GhGPX4; GhGPX5; GhGPX6; GhGPX7; GhGPX8; GhGPX9; GhGPX10; GhGPX11; GhGPX12; GhGPX13; GhGPX14; GhGPX15; GhGPX16 |
|  | GO:0009725 | response to hormone | GhGPX3; GhGPX6; GhGPX11; GhGPX14 |
|  | GO:0048608 | reproductive structure development | GhGPX2; GhGPX5; GhGPX10; GhGPX13 |
|  | GO:0061458 | reproductive system development | GhGPX2; GhGPX5; GhGPX10; GhGPX13 |
|  | GO:0009605 | response to external stimulus | GhGPX3; GhGPX6; GhGPX11; GhGPX14 |
|  | GO:0009719 | response to endogenous stimulus | GhGPX3; GhGPX6; GhGPX11; GhGPX14 |
|  | GO:0044702 | single organism reproductive process | GhGPX2; GhGPX5; GhGPX10; GhGPX13 |
|  | GO:0003006 | developmental process involved in reproduction | GhGPX2; GhGPX5; GhGPX10; GhGPX13 |
|  | GO:1901700 | response to oxygen-containing compound | GhGPX3; GhGPX6; GhGPX11; GhGPX14 |
|  | GO:0009791 | post-embryonic development | GhGPX2; GhGPX5; GhGPX10; GhGPX13 |
|  | GO:0022414 | reproductive process | GhGPX2; GhGPX5; GhGPX10; GhGPX13 |
|  | GO:0000003 | reproduction | GhGPX2; GhGPX5; GhGPX10; GhGPX13 |
|  | GO:0007165 | signal transduction | GhGPX3; GhGPX6; GhGPX11; GhGPX14 |
|  | GO:0044700 | single organism signaling | GhGPX3; GhGPX6; GhGPX11; GhGPX14 |
|  | GO:0023052 | signaling | GhGPX3; GhGPX6; GhGPX11; GhGPX14 |
|  | GO:0010033 | response to organic substance | GhGPX3; GhGPX6; GhGPX11; GhGPX14 |
|  | GO:0048731 | system development | GhGPX2; GhGPX5; GhGPX10; GhGPX13 |
|  | GO:0007154 | cell communication | GhGPX3; GhGPX6; GhGPX11; GhGPX14 |
|  | GO:0051716 | cellular response to stimulus | GhGPX3; GhGPX6; GhGPX11; GhGPX14 |
|  | GO:0007275 | multicellular organism development | GhGPX2; GhGPX5; GhGPX10; GhGPX13 |
|  | GO:0044707 | single-multicellular organism process | GhGPX2; GhGPX5; GhGPX10; GhGPX13 |
|  | GO:0048856 | anatomical structure development | GhGPX2; GhGPX5; GhGPX10; GhGPX13 |
|  | GO:0032501 | multicellular organismal process | GhGPX2; GhGPX5; GhGPX10; GhGPX13 |
|  | GO:0044767 | single-organism developmental process | GhGPX2; GhGPX5; GhGPX10; GhGPX13 |
|  | GO:0032502 | developmental process | GhGPX2; GhGPX5; GhGPX10; GhGPX13 |
|  | GO:0050794 | regulation of cellular process | GhGPX3; GhGPX6; GhGPX11; GhGPX14 |
|  | GO:0050789 | regulation of biological process | GhGPX3; GhGPX6; GhGPX11; GhGPX14 |
|  | GO:0065007 | biological regulation | GhGPX3; GhGPX6; GhGPX11; GhGPX14 |
|  | GO:0044763 | single-organism cellular process | GhGPX3; GhGPX6; GhGPX8; GhGPX11; GhGPX14; GhGPX16 |
|  | GO:0044237 | cellular metabolic process | GhGPX3; GhGPX6; GhGPX8; GhGPX11; GhGPX14; GhGPX16 |
|  | GO:0009987 | cellular process | GhGPX3; GhGPX6; GhGPX8; GhGPX11; GhGPX14; GhGPX16 |
|  | GO:0008150 | biological_process | GhGPX1; GhGPX2; GhGPX3; GhGPX4; GhGPX5; GhGPX6; GhGPX7; GhGPX8; GhGPX9; GhGPX10; GhGPX11; GhGPX12; GhGPX13; GhGPX14; GhGPX15; GhGPX16 |
| **Molecular Function (MF)** | GO:0004602 | glutathione peroxidase activity | GhGPX1; GhGPX2; GhGPX3; GhGPX4; GhGPX5; GhGPX6; GhGPX7; GhGPX8; GhGPX9; GhGPX10; GhGPX11; GhGPX12; GhGPX13; GhGPX14; GhGPX15; GhGPX16 |
|  | GO:0004601 | peroxidase activity | GhGPX1; GhGPX2; GhGPX3; GhGPX4; GhGPX5; GhGPX6; GhGPX7; GhGPX8; GhGPX9; GhGPX10; GhGPX11; GhGPX12; GhGPX13; GhGPX14; GhGPX15; GhGPX16 |
|  | GO:0016684 | oxidoreductase activity, acting on peroxide as acceptor | GhGPX1; GhGPX2; GhGPX3; GhGPX4; GhGPX5; GhGPX6; GhGPX7; GhGPX8; GhGPX9; GhGPX10; GhGPX11; GhGPX12; GhGPX13; GhGPX14; GhGPX15; GhGPX16 |
|  | GO:0016209 | antioxidant activity | GhGPX1; GhGPX2; GhGPX3; GhGPX4; GhGPX5; GhGPX6; GhGPX7; GhGPX8; GhGPX9; GhGPX10; GhGPX11; GhGPX12; GhGPX13; GhGPX14; GhGPX15; GhGPX16 |
|  | GO:0016491 | oxidoreductase activity | GhGPX1; GhGPX2; GhGPX3; GhGPX4; GhGPX5; GhGPX6; GhGPX7; GhGPX8; GhGPX9; GhGPX10; GhGPX11; GhGPX12; GhGPX13; GhGPX14; GhGPX15; GhGPX16 |
|  | GO:0003824 | catalytic activity | GhGPX1; GhGPX2; GhGPX3; GhGPX4; GhGPX5; GhGPX6; GhGPX7; GhGPX8; GhGPX9; GhGPX10; GhGPX11; GhGPX12; GhGPX13; GhGPX14; GhGPX15; GhGPX16 |
|  | GO:0046872 | metal ion binding | GhGPX8; GhGPX16 |
|  | GO:0043169 | cation binding | GhGPX8; GhGPX16 |
|  | GO:0043167 | ion binding | GhGPX8; GhGPX16 |
|  | GO:0005488 | binding | GhGPX8; GhGPX16 |
|  | GO:0003674 | molecular_function | GhGPX1; GhGPX2; GhGPX3; GhGPX4; GhGPX5; GhGPX6; GhGPX7; GhGPX8; GhGPX9; GhGPX10; GhGPX11; GhGPX12; GhGPX13; GhGPX14; GhGPX15; GhGPX16 |
| **Cellular Component (CC)** | GO:0005886 | plasma membrane | GhGPX2; GhGPX3; GhGPX4; GhGPX5; GhGPX6; GhGPX7; GhGPX10; GhGPX11; GhGPX12; GhGPX13; GhGPX14; GhGPX15 |
|  | GO:0071944 | cell periphery | GhGPX2; GhGPX3; GhGPX4; GhGPX5; GhGPX6; GhGPX7; GhGPX10; GhGPX11; GhGPX12; GhGPX13; GhGPX14; GhGPX15 |
|  | GO:0005829 | cytosol | GhGPX3; GhGPX6; GhGPX8; GhGPX11; GhGPX14; GhGPX16 |
|  | GO:0016020 | membrane | GhGPX2; GhGPX3; GhGPX4; GhGPX5; GhGPX6; GhGPX7; GhGPX10; GhGPX11; GhGPX12; GhGPX13; GhGPX14; GhGPX15 |
|  | GO:0005623 | cell | GhGPX2; GhGPX3; GhGPX4; GhGPX5; GhGPX6; GhGPX7; GhGPX8; GhGPX10; GhGPX11; GhGPX12; GhGPX13; GhGPX14; GhGPX15; GhGPX16 |
|  | GO:0044464 | cell part | GhGPX2; GhGPX3; GhGPX4; GhGPX5; GhGPX6; GhGPX7; GhGPX8; GhGPX10; GhGPX11; GhGPX12; GhGPX13; GhGPX14; GhGPX15; GhGPX16 |
|  | GO:0044444 | cytoplasmic part | GhGPX3; GhGPX4; GhGPX6; GhGPX7; GhGPX8; GhGPX11; GhGPX12; GhGPX14; GhGPX15; GhGPX16 |
|  | GO:0005737 | cytoplasm | GhGPX3; GhGPX4; GhGPX6; GhGPX7; GhGPX8; GhGPX11; GhGPX12; GhGPX14; GhGPX15; GhGPX16 |
|  | GO:0009507 | chloroplast | GhGPX4; GhGPX7; GhGPX12; GhGPX15 |
|  | GO:0009536 | plastid | GhGPX4; GhGPX7; GhGPX12; GhGPX15 |
|  | GO:0044424 | intracellular part | GhGPX3; GhGPX4; GhGPX6; GhGPX7; GhGPX8; GhGPX11; GhGPX12; GhGPX14; GhGPX15; GhGPX16 |
|  | GO:0005622 | intracellular | GhGPX3; GhGPX4; GhGPX6; GhGPX7; GhGPX8; GhGPX11; GhGPX12; GhGPX14; GhGPX15; GhGPX16 |
|  | GO:0005634 | nucleus | GhGPX8; GhGPX16 |
|  | GO:0043231 | intracellular membrane-bounded organelle | GhGPX4; GhGPX7; GhGPX8; GhGPX12; GhGPX15; GhGPX16 |
|  | GO:0043227 | membrane-bounded organelle | GhGPX4; GhGPX7; GhGPX8; GhGPX12; GhGPX15; GhGPX16 |
|  | GO:0043229 | intracellular organelle | GhGPX4; GhGPX7; GhGPX8; GhGPX12; GhGPX15; GhGPX16 |
|  | GO:0043226 | organelle | GhGPX4; GhGPX7; GhGPX8; GhGPX12; GhGPX15; GhGPX16 |
|  | GO:0005575 | cellular_component | GhGPX2; GhGPX3; GhGPX4; GhGPX5; GhGPX6; GhGPX7; GhGPX8; GhGPX10; GhGPX11; GhGPX12; GhGPX13; GhGPX14; GhGPX15; GhGPX16 |

**Table S6.** KEGG enrichment analysis.

| **Pathway** | **Pathway ID** | **Genes** |
| --- | --- | --- |
| **Arachidonic acid metabolism** | ko00590 | GhGPX7; GhGPX8; GhGPX9; GhGPX10; GhGPX11; GhGPX12; GhGPX13; GhGPX14; GhGPX15; GhGPX16; GhGPX1; GhGPX2; GhGPX3; GhGPX4; GhGPX5; GhGPX6 |
| **Glutathione metabolism** | ko00480 | GhGPX7; GhGPX8; GhGPX9; GhGPX10; GhGPX11; GhGPX12; GhGPX13; GhGPX14; GhGPX15; GhGPX16; GhGPX1; GhGPX2; GhGPX3; GhGPX4; GhGPX5; GhGPX6 |
| **Metabolic pathways** | ko01100 | GhGPX7; GhGPX8; GhGPX9; GhGPX10; GhGPX11; GhGPX12; GhGPX13; GhGPX14; GhGPX15; GhGPX16; GhGPX1; GhGPX2; GhGPX3; GhGPX4; GhGPX5; GhGPX6 |

**Table S7. The KaKs analysis of *GhGPXs* gene family.**

| **Seq_1** | **Seq_2** | **Ka** | **Ks** | **Ka_Ks** |
| --- | --- | --- | --- | --- |
| GhGPX1 | GhGPX9 | 0.0247 | 0.0578 | 0.4273 |
| GhGPX2 | GhGPX5 | 0.1595 | 0.6636 | 0.2404 |
| GhGPX3 | GhGPX4 | 0.2029 | NaN | NaN |
| GhGPX4 | GhGPX7 | 0.0542 | 0.5129 | 0.1056 |
| GhGPX4 | GhGPX12 | 0.0422 | 0.0748 | 0.5642 |
| GhGPX5 | GhGPX13 | 0.0075 | 0.0270 | 0.2789 |
| GhGPX2 | GhGPX10 | 0.1187 | 0.2166 | 0.5482 |
| GhGPX3 | GhGPX11 | 0.0188 | 0.0518 | 0.3636 |
| GhGPX4 | GhGPX11 | 0.2027 | NaN | NaN |
| GhGPX5 | GhGPX10 | 0.0852 | 0.5673 | 0.1501 |
| GhGPX2 | GhGPX13 | 0.1669 | 0.6355 | 0.2626 |
| GhGPX4 | GhGPX15 | 0.0465 | 0.4892 | 0.0951 |
| GhGPX6 | GhGPX14 | 0.0153 | 0.0332 | 0.4599 |
| GhGPX7 | GhGPX12 | 0.1343 | 0.5665 | 0.2371 |
| GhGPX7 | GhGPX15 | 0.0180 | 0.0328 | 0.5481 |
| GhGPX10 | GhGPX13 | 0.0853 | 0.5898 | 0.1446 |
| GhGPX12 | GhGPX15 | 0.1321 | 0.5638 | 0.2344 |

**Table S8. Interaction protein of GhGPX4.**

| **Protein** | **Name** | **Description** | **Coverage (%)** | **MV (kDa)** |
| --- | --- | --- | --- | --- |
| **1** | **GhVATE** | V-type proton ATPase subunit E | 72 | 27.176 |
| **2** | **GhAPX2** | L-ascorbate peroxidase 2 | 66 | 27.562 |
| **3** | **GhGAPC2** | Glyceraldehyde-3-phosphate dehydrogenase 2 | 54 | 36.563 |
| **4** | **GhGSTF6** | Glutathione S-transferase F6 | 48 | 24.086 |
| **5** | **GhDHAR2** | Glutathione S-transferase DHAR2 | 45 | 23.5 |
| **6** | **GhMDHAR5** | Monodehydroascorbate reductase 5 | 38 | 52.813 |
